# Ecosystem Interactions Underlie the Spread of Avian Influenza A Viruses with Pandemic Potential

**DOI:** 10.1371/journal.ppat.1005620

**Published:** 2016-05-11

**Authors:** Justin Bahl, Truc T. Pham, Nichola J. Hill, Islam T. M. Hussein, Eric J. Ma, Bernard C. Easterday, Rebecca A. Halpin, Timothy B. Stockwell, David E. Wentworth, Ghazi Kayali, Scott Krauss, Stacey Schultz-Cherry, Robert G. Webster, Richard J. Webby, Michael D. Swartz, Gavin J. D. Smith, Jonathan A. Runstadler

**Affiliations:** 1 Center for Infectious Diseases, The University of Texas School of Public Health, Houston, Texas, United States of America; 2 Program in Emerging Infectious Diseases, Duke-National University of Singapore Graduate Medical School, Singapore, Singapore; 3 Division of Comparative Medicine, Department of Biological Engineering, Massachusetts Institute of Technology, Cambridge, Massachusetts, United States of America; 4 Department of Pathobiological Sciences, School of Veterinary Medicine, University of Wisconsin-Madison, Madison, Wisconsin, United States of America; 5 J. Craig Venter Institute, Rockville, Maryland, United States of America; 6 Department of Infectious Diseases, St. Jude Children's Research Hospital, Memphis, Tennessee, United States of America; 7 Duke Global Health Institute, Duke University, Durham, North Carolina, United States of America; Imperial College London, UNITED KINGDOM

## Abstract

Despite evidence for avian influenza A virus (AIV) transmission between wild and domestic ecosystems, the roles of bird migration and poultry trade in the spread of viruses remain enigmatic. In this study, we integrate ecosystem interactions into a phylogeographic model to assess the contribution of wild and domestic hosts to AIV distribution and persistence. Analysis of globally sampled AIV datasets shows frequent two-way transmission between wild and domestic ecosystems. In general, viral flow from domestic to wild bird populations was restricted to within a geographic region. In contrast, spillover from wild to domestic populations occurred both within and between regions. Wild birds mediated long-distance dispersal at intercontinental scales whereas viral spread among poultry populations was a major driver of regional spread. Viral spread between poultry flocks frequently originated from persistent lineages circulating in regions of intensive poultry production. Our analysis of long-term surveillance data demonstrates that meaningful insights can be inferred from integrating ecosystem into phylogeographic reconstructions that may be consequential for pandemic preparedness and livestock protection.

## Author Summary

It is assumed that AIV outbreaks in poultry are introduced from wild birds. To test this, we incorporated ecosystem and location of isolation into a comparative genetic analysis. We show high rates of viral transmission from domestic to wild birds within a region and, that wild birds could transmit AIV to poultry between regions. However, the highest rates of viral flow between regions was among domestic populations, indicating poultry trade may play a major role in spreading viral populations. We demonstrate that interactions between migratory birds and animal productions systems contribute to the emergence of AIV. The assumption of unidirectional viral flow from wild birds to domestic poultry provides an incomplete picture of influenza ecology and may confound control efforts.

## Introduction

Intensive agriculture has allowed AIV circulating in wild bird populations and multi-host poultry systems (domestic food birds including chicken, duck, goose, pigeon) to interact, shaping the diversity of subtypes with pandemic potential [1]. The recently emerged H7N9 viruses containing H9N2-origin internal genes highlight that co-circulation of subtypes concealed within poultry systems can enhance the pandemic threat of influenza [2]. Such interactions are not unique. Over the last decade, H9N2 viruses have donated gene segments to several virus subtypes infecting poultry and humans, including the highly pathogenic avian influenza (HPAI) H5N1 panzootic that emerged in 2003 and persists to the present day [3–5]. Transmissions across the wild-domestic bird interface and genomic reshuffling within poultry have contributed to the emergence, spread and persistence of novel H5, H6, H7, and H9 AIV genotypes, which have caused human infection [4, 6, 7]. Despite the importance of viral transmission between natural and domestic systems, the role of these interactions in determining viral diversity and distribution has not been adequately studied.

AIV transmission between wild reservoirs and domestic animals takes place where natural and agricultural ecosystems overlap, a scenario that occurs worldwide. For example, transmission between wild and domestic birds led to H6 outbreaks in Californian poultry [8]; low pathogenic (LPAI) H5 viruses from wild birds in Italy were linked to poultry disease in Asia [7, 9]; and wild bird-origin H9 viruses circulating in Korea later emerged in domestic flocks [10]. Most recently, H5 viruses detected in East Asia have spread to European and North American poultry, consistent with intercontinental migration of wild birds [11]. Although often detected in domestic birds, viral communication between populations is not one-way. In 2005, HPAI H5N1 jumped from domestic birds to infect bar-headed geese (*Anser indicus*) at Qinghai Lake, China (12). This triggered large-scale wildlife die-offs, contributed to the global spread of HPAI H5N1 virus and placed considerable burdens on government resources to mitigate global spread [12, 13].

H9 viruses are endemic in terrestrial poultry in China, the Middle East, and Europe and occur globally in a diversity of wild bird taxa [14]. Although H9 in poultry is not a notifiable disease, it is listed by the World Health Organization as a candidate for the next global pandemic along with H5 and H7 [15]. Gene segment exchange with H9 has facilitated generation of novel reassortants, including the 1997 H5N1 strain that caused human infections and fatalities in Hong Kong [3]. Furthermore, periodic human infections with H9 subtype viruses have led to intensive epidemiological surveillance of wild and domestic birds to identify the infection source [3, 6, 16, 17]. These events have prompted regular collection and sequencing of H9 subtype viruses providing a globally sampled genetic dataset where the contributions of wild and domestic animals to the global spread can be modelled using phylogeographic approaches.

Despite an expanding AIV host range, the role of ecosystem interactions (i.e. transmission between wild and domestic animals) in generating and spreading novel influenza viruses over large distances remain poorly characterized. In this study we integrate the geographical and ecological context of transmission into a statistical phylogenetic model. Using H9, H3 and H6 sequence data, including newly acquired sequences from North American wild birds, we characterize the contribution of wild and domestic birds to the global distribution of AIV and estimate the role of inter-ecosystem dynamics on viral diversity and persistence. We hypothesize that viruses circulating among wild migratory birds may connect a global network of influenza outbreaks that occur locally in domestic birds. To test this, we incorporated ecosystem and geographic location of isolation into a comparative genetic analysis of H9 hemagglutinin (HA) sequence data (see S1 Text and S1 Table). H9 subtype viruses are commonly isolated from domestic poultry and wild birds [3, 7, 18–24]. Periodic human infections have resulted in intensive surveillance of domestic birds in order to identify the sources of infection [16, 17]. As a result, a robust dataset of H9 subtype viruses is available from both domestic and wild birds throughout the world. Since poultry and wild birds infected with H9 viruses do not often manifest major disease symptoms, vaccination or active control efforts are limited for this subtype. We utilize the H9 virus surveillance and sequence reporting, enhanced with novel sequences acquired from North American wild bird surveillance collected between 1974 and 2013. We build on previous phylogeographic methods [25] to integrate wild and domestic ecosystems in order to 1) estimate an asymmetric spatial transition matrix; 2) model viral spread among wild or domestic populations; and 3) assess the relative risk of virus emergence from discrete locations and ecosystems. We extend our analysis to other avian influenza A virus subtypes (H3 and H6) in order to assess if inferences made from comparative genetic analysis of H9 gene sequences could be generalized.

## Results

### Poultry production, live animal trade and disease surveillance

**Fig 1:**
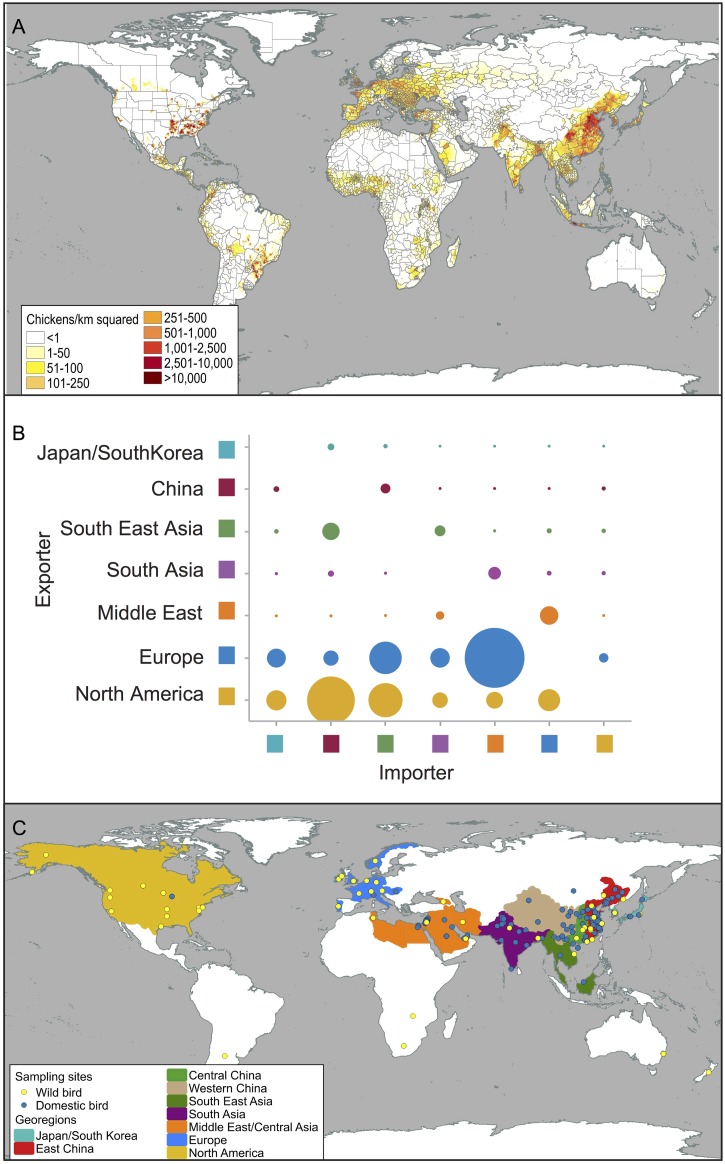
Poultry production, global trade intensity and location of viral sampling. (**A)** Map showing global chicken density (millions of chickens/km^2^) **(B)** Reported trade intensity of domestic poultry 1995 to 2011 between regions **(C)** Locations of available viral sequences within discrete regions defined by sampling effort, poultry production and sequence data.

Poultry production density and disease surveillance effort vary considerably between countries. We therefore incorporated this information in defining our discrete geographic units for our migration model. Mapping all available poultry and wild bird virus isolates allows for a qualitative assessment showing that global sampling sites correspond well with the intensity and distribution of poultry production systems, particularly in China and the Middle East (Fig 1A–1C). Samples from wild and domestic avian hosts spatially overlap. It is possible that surveillance in wild birds was in response to AIV detection in domestic birds. There are few long-term virological surveillance programs and most detection was based on opportunistic sampling [2,4,6–8,10,11]. As a result, there are many more isolates from domestic birds than wild birds, despite the importance of these hosts in the ecology, evolution and emergence of AIV [11]. Sampled sites similarly overlap with areas of intense duck production, although domestic duck rearing is far less common than chickens worldwide (S1 Fig). While we assume that the virus behaves the same in all poultry hosts, experimental data indicates this may be a poor assumption [18]. Unfortunately, limited information on virus prevalence or epidemiology in various domestic host species between countries makes it difficult to treat individual species separately, thereby necessitating the grouping used here.

We assume that if AIV is transmitted between domestic populations it is likely correlated with live poultry trade between countries (Fig 1B). We use this framework to interpret our phylogeographic reconstruction results where, we believe that the movement of viruses between discrete states is limited to the movement of live animals, whether through trade or wild animal migration. It is possible that transmission between regions could be linked to trade of animal product or other cryptic means, but for this study we have not considered other mechanisms of viral spread. According to official trade data available from the Food and Agriculture Organization of the United Nations (UN-FAO), intercontinental movement of live poultry primarily involves exports from Europe and North America into the eastern hemisphere: Middle East, China and Southeast Asia (Fig 1B). Regional flow of animals throughout Europe showed that few countries (i.e. Germany, Netherlands) were the main importers of live animals from other European nations and were also exporters to the Middle East and Southeast Asia. The majority of poultry production in China is for domestic consumption, although trade with Southeast and East Asia (Japan/South Korea) accounted for the bulk of live animal exports (Fig 1B). After the emergence and spread of HPAI H5N1, there was a marked decline in poultry exports with the majority of official trade restricted to supplying Hong Kong and Macau. Detailed trade flow and animal population data led us to subdivide China into 3 distinct regions (East, Central and West China) based on production and consumption, and to combine Japan and South Korea into a discrete isolated (particularly after 2004) geo-region relative to China (Fig 1B and 1C). Based on the distribution of available AIV sequences from poultry and global patterns of live poultry trade, we therefore identified 9 discrete regions for which viral migration patterns could be estimated (Fig 1C, S2 Fig and S2 Table).

### Global distribution of wild bird isolates

Wild bird surveillance is often conducted to identify potential virus sources following outbreaks in poultry [26]. Consequently, large overlap between wild bird and poultry surveillance sites exists (Fig 1C and S2 Table). Notable exceptions include long-term influenza surveillance studies of migratory birds in North America (Delaware Bay, Alberta) and Europe (Ottenby, Sweden) [27]. With the exception of waterfowl, most other wild bird species are opportunistically sampled rather than targeted [28]. Sampling inconsistencies across different species mean that sample sizes of virus isolates are not appropriate to infer rates of interspecies transmission among wild bird taxa (i.e. mallard, *Anas platyrhynchos* to northern pintail, *A*. *acuta*). Nevertheless, the data from wild birds was well suited for estimating viral flow between wild and domestic systems. In our analysis we assumed that the virus behaves similarly in all wild species and classified isolates as either ‘wild’ or ‘domestic’ to estimate viral transmission rate between populations.

### Phylogenetic reconstruction and source/sink dynamics

**Fig 2:**
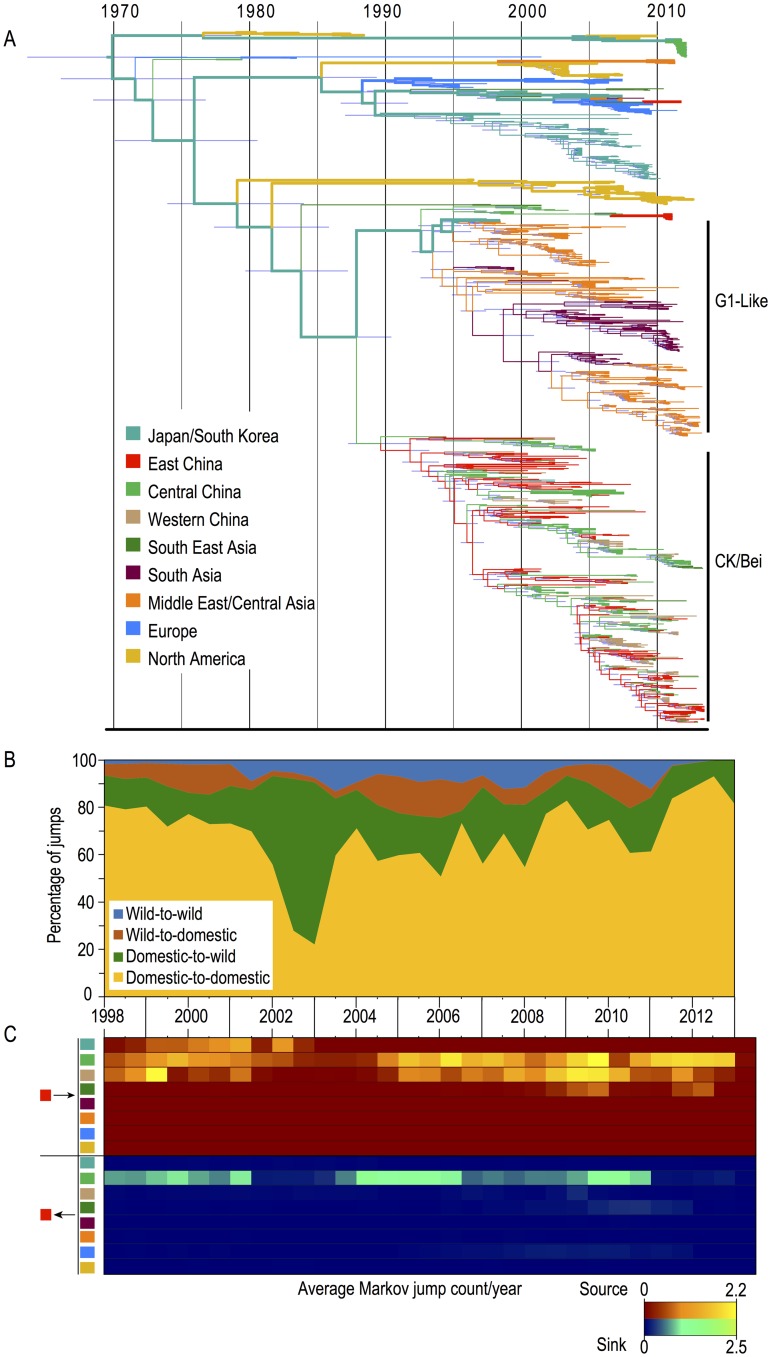
Phylogenetic estimation of ecological interactions and geographic spread. (**A)** H9-HA phylogenetic tree for global isolates where branches are coloured according to discrete geographic region and thick and thin lines indicate ancestral transitions between natural and agricultural ecosystems respectively (**B)** Graph showing the proportional Markov jump counts between ecosystems over time (**C)** Heat maps showing mean H9 migrations estimated to and from East China per year. Heat maps for other regions are shown in S4 Fig.

Phylogenetic reconstruction of the H9-HA gene showed a mixture of North American and Eurasian wild bird isolates and two recently diverged poultry lineages (G1 and Ck/Bei lineages; Fig 2A and S3 Fig), consistent with previous studies [3, 29–31]. Our analysis demonstrated that the H9 lineage was younger and less geographically structured than other HA subtypes [8, 32, 33]. The estimated tree root age from sampled H9 strains revealed an origin between 1964 and 1975, suggesting a recent selective sweep removed genetic signals of long-term geographic structuring from the population. Periodic sweeps have been implicated in lasting changes in the viral population of multiple subtypes [33, 34].

We estimated the ancestral locations of isolates collected from the 9 discrete geographic regions defined above (Fig 2A) and stratified the observations as viruses collected from either wild or domestic birds. We found frequent viral flow among regions and identified East/Central China (Ck/Bei lineage) and the Middle East (G1 lineage) as the primary sources for H9 virus spread among poultry (Fig 2B and Table 1). We determined the number of transitions emerging from each ecosystem state from 1998 through 2013, and show that the viral population was primarily emerging from domestic populations with most transitions into other domestic populations (Fig 2B). Approximately 90% of all transitions emerged from the domestic populations. The mean waiting time in each state as a proportion of the total phylogenetic tree time show that the populations was primarily circulating among poultry located in East/Central China and the Middle East (Table 1). Date estimates suggested these two regional populations diverged around 1988 (95% Bayesian Credible Interval: BCI 1985–1990), coinciding with industrialization of poultry production in Asia [35]. Our results also suggest that both CK/Bei and G1 lineages emerged from independent wild bird introductions. Over the last decade these regions have acted as two distinct and persistent gene pools (Fig 2B and Table 1), reflecting the establishment of East Asia and the Middle East as independent centers of poultry production [14].

**Table 1:** Mean waiting times for the H9 population in each state calculated as a proportion of the total branch times across the phylogenetic tree sampled per MCMC*.

	Ecotype	
	Domestic	Wild
Japan/Korea	0.078 (0.069, 0.093)	0.046 (0.027, 0.089)
E China	0.196 (0.171, 0.229)	0.005 (0.002, 0.013)
C China	0.153 (0.124, 0.183)	0.012 (0.009, 0.019)
W China	0.047 (0.039, 0.058)	-
SE Asia	0.023 (0.015, 0.035)	0.000 (0, 0.007)
S Asia	0.086 (0.078, 0.095)	0.001 (0, 0.008)
Middle East	0.187 (0.177, 0.200)	0.013 (0.007, 0.023)
Europe	0.022 (0.008, 0.033)	0.029 (0.02, 0.044)
N America	-	0.101 (0.062, 0.128)

*calculated from the final 1000 sampled MCMC steps. Mean values and 99% BCI are presented in the brackets.

In our analysis we used a non-reversible model such that the direction of migration could be inferred to assess whether a region acted as a source or a sink from 1998 through 2013 [25, 36, 37]. For each location, we averaged the number of state changes observed/year for trees sampled from the posterior distribution. Even though East China was a persistent source population, the transmission was primarily limited to within China. East China primarily receives viruses from Central China and vice versa (Fig 2C and S4 Fig). Prior to 2003 there was some exchange with Japan. The rapid disruption in viral flow between these regions corresponded with restrictions on live poultry exports after the emergence and regional spread of highly pathogenic avian influenza H5N1 [35]. The Middle East region was primarily a sink for G1 lineage viruses originating in South Asia and Europe (S4 Fig). Transmission from the Middle East to South Asia and Europe, although rare, was evident in other lineages (S4 Fig).

Our results showed enhanced risk of viral emergence from East China, whereas Western China and Southeast Asia represent regions with enhanced risk of receiving H9 viruses (Table 2). By evaluating the relative risk for regional source/sink transmission from domestic or wild birds, we see enhanced risk for transmission originating from wild birds in Japan/South Korea and Europe. In contrast, wild birds from the Middle East, South Asia, East and Central China were more likely to receive viruses. There was enhanced risk for domestic birds in East China, South Asia and the Middle East to be a source for transmission of H9 viruses. Domestic birds in Japan/S Korea, Western China, Southeast Asia and Europe were more likely to be sinks (Table 2).

**Table 2:** Mean relative risk (RR) ratios for each region to act as a global source or sink population estimated from the total number of Markov jumps from or to each discrete state.

	Overall RR*	RR by Ecotype	
		Domestic	Wild
Japan/Korea	0.94 (0.36, 1.50)	**0.18 (0.12, 0.53)**	8.30^†^ (1.00, 15.87)
E China	**3.08 (1.64, 5.23)**	**4.06 (1.80, 8.59)**	**0.12 (0.00, 0.76)**
C China	1.12 (0.59, 1.80)	1.19 (0.58, 2.14)	**0.49 (0.35, 0.89)**
W China	**0.04 (0.00, 0.21)**	**0.04 (0.00, 0.18)**	-
SE Asia	**0.17 (0.00, 0.40)**	**0.13 (0, 0.38)**	0.59 (0.00, 2.33)
S Asia	1.23 (0.57, 2.00)	2.12^†^ (0.68, 5.19)	0.05^†^ (0.00, 1.06)
Middle East	0.92 (0.73, 1.23)	**2.30 (1.34, 5.87)**	**0.49 (0.17, 0.89)**
Europe	1.01 (0.60, 2.00)	**0.04 (0.00, 0.57)**	**6.75 (2.63, 11.65)**
N America	6.12 (0.00, 50.00)	-	3.89 (0.00, 22.00)

Bolded values indicate those regions where the 99% BCI does not cross 1 indicating significance. RR values >1 indicate source region; <1 indicate sink region.

*Overall RR combines domestic and wild bird transitions.

^†^ Indicates where the 95% BCI does not cross 1.

### Migration patterns and ecosystem interactions

**Fig 3:**
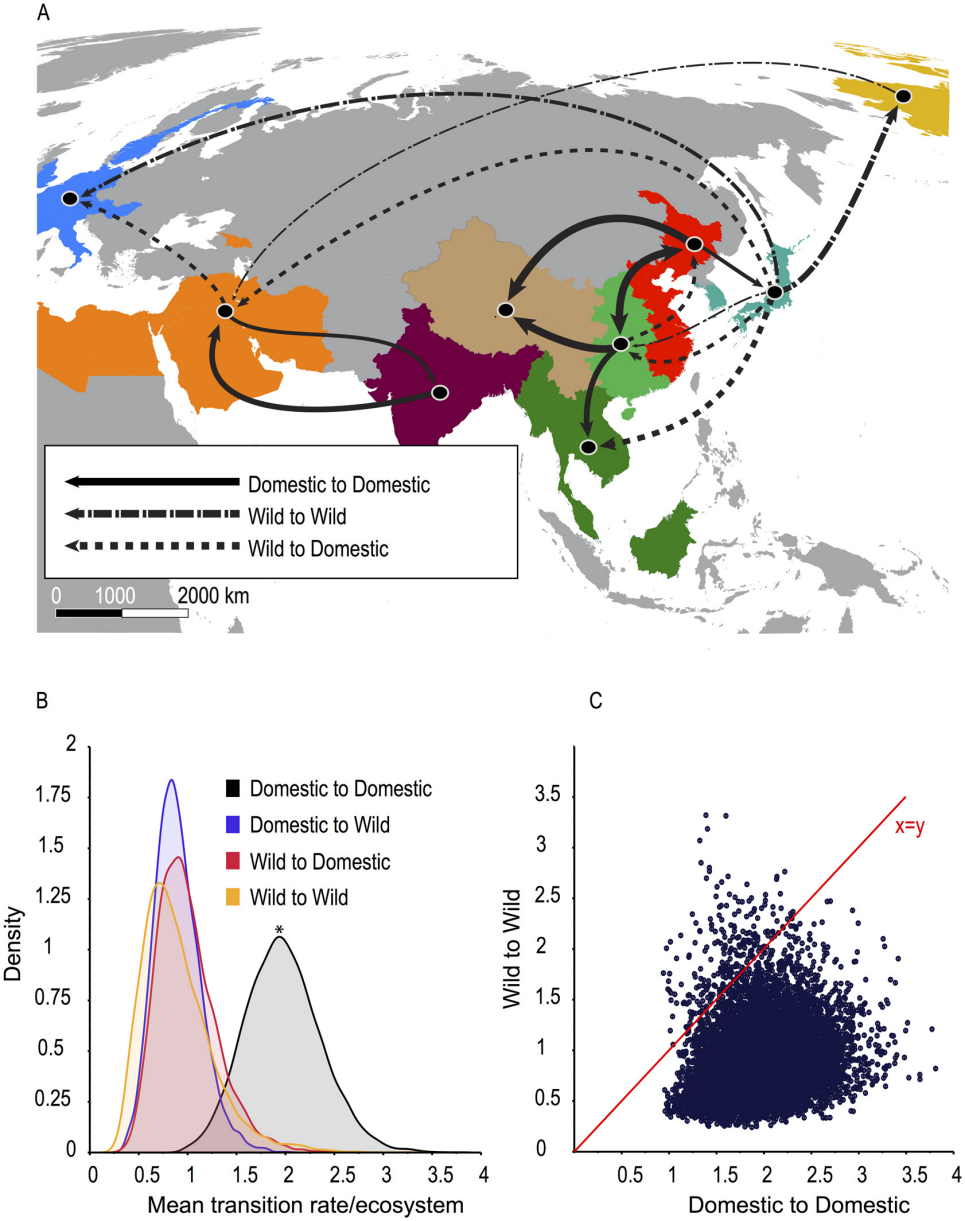
Inferred migration rates and patterns. **(A)** Map showing statistically supported transitions between geographic regions by ecosystem. Line thickness corresponds to viral flow rates shown in Table 2 (thinnest<0.5; ≥0.5<1; ≥1<2;≥ 2 thickest). (**B)** Density distribution of statistically supported mean transition rates between ecosystems. *Domestic-to-domestic rates are significantly faster than domestic-to-wild (BF>100), wild-to-domestic (BF = 62.3), and wild-to-wild (BF = 39.4). (**C)** Statistically supported mean migration rates per MCMC step of wild-to-wild avian transitions versus domestic-to-domestic avian transitions.

Domestic-to-wild virus communications were restricted to within regions, whereas wild-to-domestic transmissions occurred both within and between regions (Table 3). Two-way viral flow between ecosystems occurred within China and the Middle East, emphasizing the importance of these regions for ecosystem interactions (Table 3). Domestic-to-domestic transmission was between adjacent regions with no significant long distance migrations (Fig 3A). In contrast, supported wild-to-wild transmissions occurred over long and short distances (Fig 3A). Our results suggest that poultry systems have provided a persistent source for regional H9 spread, whereas wild bird-mediated dispersal provided a mechanism for both intercontinental (Japan/S Korea-North America and Japan/S Korea-Europe) and regional spread (East Asia-Southeast Asia) along known waterbird migratory routes [38, 39] (Fig 3A). Despite strong statistical support for these migration events, the importance of Japan and South Korea for long distance wild bird carriage of viruses is difficult to assess due to sparseness of wild bird sampling in this region (Fig 2A). We also found strong support for wild-bird mediated viral migration between North America and the Middle East (Table 3). In both locations, viruses were isolated from gulls and shorebirds. In our reconstruction there was more than 10 years of under-sampled diversity between viruses in gulls from the Republic of Georgia and the putative North American ancestor. It is unlikely that this was a direct transmission between populations. The most parsimonious explanation is that this virus transmitted among gulls during this period, implying that persistence in wild bird populations may contribute to the long-distance spread of H9 viruses.

**Table 3:** Mean migration rates between avian ecosystems across sampling locations. Statistically supported interactions are shown in bold. Italicized text indicates interactions between ecosystems.

			Destination															
			Domestic								Wild							
			Japan/ S Korea	E China	C China	W China	SE Asia	S Asia	Middle East	Europe	Japan/ S Korea	E China	C China	SE Asia	S Asia	Middle East	Europe	North America
**Origin**	**Domestic**	Japan/ S Korea	-	0.26	0.28	0.26	0.28	0.22	0.23	0.23	***0*.*42*** ^§^ ***[0*.*01*, *1*.*01]***	*0*.*24*	*0*.*19*	*0*.*24*	*0*.*2*	*0*.*22*	*0*.*2*	*0*.*33*
		E China	**0.98** ^§^ **[0.32, 1.77]**	-	**5.15** ^§^ **[2.54, 7.73]**	**2.38** ^§^ **[0.97, 3.91]**	0.38	0.12	0.25	0.13	*0*.*14*	***0*.*72*** ^§^ ***[0*.*17*, *1*.*39]***	*0*.*23*	*0*.*24*	*0*.*1*	*0*.*13*	*0*.*13*	*0*.*15*
		C China	0.46	**2.63** ^§^ **[0.77, 4.65]**	-	**2.52** ^§^ **[1.01, 4.24]**	**1.11** ^§^ **[0.28, 2.09]**	0.28	0.32	0.25	*0*.*22*	*0*.*33*	***1*.*58*** ^§^ ***[0*.*55*, *2*.*71]***	*0*.*29*	*0*.*1*	*0*.*19*	*0*.*12*	*0*.*15*
		W China	0.32	0.53	0.61	-	0.49	0.26	0.36	0.30	*0*.*27*	*0*.*33*	*0*.*36*	*0*.*38*	*0*.*26*	*0*.*24*	*0*.*28*	*0*.*28*
		SE Asia	0.49	0.42	0.41	0.38	-	0.32	0.36	0.32	*0*.*35*	*0*.*67*	*0*.*42*	***0*.*62*** ^ǂ^ ***[4E-3*, *1*.*45]***	*0*.*29*	*0*.*31*	*0*.*34*	*0*.*25*
		S Asia	0.20	0.18	0.25	0.19	0.17	-	**1.17** ^§^ **[0.14, 2.57]**	0.22	*0*.*41*	*0*.*17*	*0*.*17*	*0*.*29*	***0*.*58*** ^§^ ***[0*.*06*, *1*.*28]***	*0*.*33*	*0*.*21*	*0*.*21*
		Middle East	0.18	0.12	0.1	0.13	0.12	**0.61** ^†^ **[0.08, 1.22]**	-	0.31	*0*.*4*	*0*.*17*	*0*.*15*	*0*.*12*	*0*.*16*	***1*.*46*** ^§^ ***[0*.*52*, *2*.*47]***	*0*.*2*	*0*.*15*
		Europe	0.43	0.27	0.36	0.32	0.39	0.42	0.40	-	*0*.*42*	*0*.*48*	*0*.*37*	*0*.*3*	*0*.*31*	*0*.*74*	***0*.*76*** ^†^ ***[0*.*01*, *1*.*89]***	*0*.*31*
	**Wild**	Japan/ S Korea	***0*.*44*** ^†^ ***[0*.*01*, *1*.*07]***	*0*.*7*	***0*.*97*** ^ǂ^ ***[0*.*14*, *2*.*02]***	*0*.*23*	***1*.*01*** ^§^ ***[0*.*16*, *2*.*00]***	*0*.*55*	***0*.*79*** ^†^ ***[0*.*03*, *1*.*74]***	*0*.*6*	-	0.32	**0.45** ^†^ **[1E-3, 1.08]**	0.21	0.16	0.56	**0.85** ^§^ **[0.11, 1.83]**	**1.31** ^ǂ^ **[0.38, 2.53]**
		E China	*0*.*39*	*0*.*5*	*0*.*41*	*0*.*4*	*0*.*69*	*0*.*37*	*0*.*36*	*0*.*38*	0.34	-	0.34	0.45	0.4	0.38	0.34	0.33
		C China	*0*.*38*	***0*.*99*** ^ǂ^ ***[0*.*05*, *2*.*25]***	***0*.*97*** ^†^ ***[0*.*02*, *2*.*25]***	*0*.*35*	*0*.*41*	*0*.*44*	*0*.*46*	*0*.*38*	0.44	0.47	-	0.38	0.31	0.39	0.34	0.37
		SE Asia	*0*.*38*	*0*.*34*	*0*.*35*	*0*.*37*	*0*.*61*	*0*.*42*	*0*.*35*	*0*.*4*	0.39	0.4	0.35	-	0.41	0.34	0.32	0.36
		S Asia	*0*.*35*	*0*.*31*	*0*.*37*	*0*.*31*	*0*.*36*	*0*.*45*	*0*.*33*	*0*.*38*	0.38	0.37	0.43	0.36	-	0.38	0.36	0.36
		Middle East	*0*.*4*	*0*.*38*	*0*.*33*	*0*.*3*	*0*.*36*	*0*.*88*	*0*.*81*	***0*.*98*** ^§^ ***[0*.*08*, *2*.*24]***	0.65	0.46	0.36	0.35	0.41	-	0.42	0.42
		Europe	*0*.*4*	*0*.*41*	*0*.*46*	*0*.*35*	*0*.*54*	*0*.*56*	*0*.*61*	***1*.*37*** ^§^ ***[0*.*08*, *2*.*89]***	0.52	0.53	0.33	0.35	0.31	0.69	-	0.42
		North America	*0*.*4*	*0*.*33*	*0*.*46*	*0*.*26*	*0*.*22*	*0*.*24*	*0*.*46*	*0*.*39*	0.93	0.3	0.32	0.2	0.31	**0.45** ^†^ **[0.02, 1.09]**	0.48	-

BSSV statistically supported migration rates with 95% Bayesian credible intervals (BCI), where the Bayes factor was >14.

^†^14 > BF ≥ 30;

^ǂ^30 > BF ≥ 100;

^§^ BF > 100.

Integrating ecosystem and region into our migration model provided the most comprehensive description of global H9 distribution and diversity. Domestic-to-domestic transitions among regions, especially between East China and Central China, were significantly faster than all other estimated migration patterns, suggesting poultry trade was likely responsible for spreading H9 subtype virus (Fig 3B and 3C). This trend was also reflected at the global scale, whereby migration rates among domestic birds was significantly higher than among wild birds and between ecosystems (wild-to-domestic or domestic-to-wild) (Fig 3).

We chose to extend our model with analysis of AIV subtypes H3 and H6 HA gene sequences. Similar diffusion patterns (i.e. ecosystem transition patterns or rates) between subtypes may suggest similar underlying processes. Analysis of H3 subtype HA gene sequences showed similar results to those described above for H9 (S5 Fig, S6 Fig and S3 Table). In contrast, the role of virus ecosystem interactions in determining viral distribution of H6 subtype viruses was less clear, even though similar transmission patterns were observed (S7 Fig, S4 Table). Despite evidence for two-way transmission of H6 viruses between wild or domestic populations (S8 Fig and S4 Table), our analysis shows no support for either ecosystem playing a larger role in the distribution of these viruses (see S1 Text).

Our model for ancestral state reconstruction was restricted to locations and ecosystems where viruses were observed, which results in an inherent bias in the reconstruction of migration or ecosystem transitions. For example, in our analysis of H9 subtype virus, wild bird isolates were not observed from western China and therefore, this state was not represented in our ancestral state reconstruction. While it is possible that the virus population did spend time circulating among wild birds in western China, the lack of contemporary observations imposes limitations on the ancestral state reconstruction approach taken in this study. Similarly, North American H9 isolates from domestic birds were not present in our reconstruction of migration patterns and ecosystem interactions. To investigate if our ancestral reconstruction was sensitive to sampling bias, we randomized the location assignments at the tips throughout the MCMC procedure to determine if the posterior transition rate estimate and root state probability converged on the expected prior under the sampling scenario we used, as well as randomly generated alternatives [37]. For each subtype analyzed the posterior empirical frequency converged to the prior root state probability for all sampling scenarios (S9 Fig). In addition, the ecosystem transition rate estimates converged on the prior expectation where all rates were approximately equal (S10, S11 and S12 Figs). Despite uneven sampling of domestic and wild populations these analyses suggest that signals of ecosystem/spatial structure in the data inform our estimates and were not biased by the sampling scheme.

## Discussion

Our findings highlight that transmission among domestic flocks drove the majority of H9 dispersal to adjacent regions. Generally, wild bird migrations provide opportunities for the widespread movement of viruses with pandemic potential, but this did not play a greater role in viral spread than transmission among poultry populations, despite frequent transmission between wild and domestic birds. The most significant contribution from wild birds to emerging AIV ecology is the redistribution of gene segments over large distances, thereby increasing biodiversity and creating opportunities for novel variants to emerge. Failure to control H9 outbreaks in domestic populations can therefore contribute to the emergence and spread of new influenza variants [20]. Our results suggest that viral flow between wild and domestic systems contributes to the persistence, and spread of AIV.

The continued circulation of AIV in domestic populations poses a public health risk [2]. In this study, we capitalized on the robust surveillance of H9 viruses among avian populations to elucidate the role of viral transmission between wild and domestic ecosystems in the global spread and persistence of AIV. Parallels exist between the mechanisms of H9N2 global dispersal uncovered here and other subtypes. Most notably, transmissions of HPAI H5N1, and recently HPAI H5N8, have been perpetuated and spread by both wild bird migration and domestic poultry trade [13, 40, 41]. The complex interactions characterizing these systems have contributed to the genetic diversity and widespread diffusion of these viruses throughout Asia, Africa, Europe, and most recently, North America [4, 11].

Similar models may be extended to understand the mechanisms of spread and emergence of other influenza subtypes, although the paucity of surveillance data may be a limiting factor. While analysis of H3 subtype virus HA gene sequences supported our findings that transitions among domestic populations may be driving the spread of influenza viruses, our analysis of H6 viruses was less clear. H6 subtype viruses were prevalent in domestic ducks in southern China [24]. While 75% of the global duck production (including reared wild ducks) occurs in China [42] production is primarily for domestic consumption, with Hong Kong as the largest importer of duck meat [35]. Even though the large-scale transmission patterns were similar across subtypes, the binning of discrete geographic states used in our analysis of H6 could not capture the majority of domestic trade within China. It is likely that alternative sampling strategies are necessary to investigate the role of ecosystems interactions and poultry trade in maintaining H6 subtype populations.

Production systems that promote the two-way transmission of viruses between wild and domestic avian hosts facilitate the generation of potentially pandemic AIV and may lead to widespread outbreaks that are difficult to contain. Surveillance programs focused on detecting highly pathogenic subtypes in symptomatic poultry falls short of identifying the mechanisms of emergence, spread and genomic reshuffling. Active systematic surveillance for AIV in both wild and domestic populations allow for the continued development of models needed to test the role of various species or populations in viral persistence. A limitation of this study is that reconstruction of viral movement patterns was limited to transmission among the populations surveyed. It is likely that unsampled populations play a role in the spread and persistence of AIV. Systematic surveillance programs are critical to assess the risk of disease emergence and spread of AIV by wild migratory birds. Analysis of long-term surveillance data enables meaningful insights necessary to develop appropriately informed predictive models. Inferences from such models are consequential for pandemic preparedness and livestock protection.

## Materials and Methods

### Poultry trade relationships

Using available census data for chicken and ducks we produced heat maps showing poultry production intensity. Mapping all available H9-HA sequence data on top of the heat maps allows us to visually assess the distribution of sampling and production regions. This assessment was used to determine appropriate geographic regions to incorporate into our model and identify regions with few samples. Data on the international trade of live poultry from 1995–2011 (the most current year of data available) was downloaded from the United Nations, Food & Agriculture Organization (UN-FAO: faostat3.fao.org, accessed June 11, 2015). The quantity of chickens and ducks traded was chosen as the metric to assess trade relationships. All years were summed to generate long-term estimates of international trade and countries were aggregated into 7 regions: Japan/South Korea, China, South East Asia, South Asia, Middle East, Europe and North America, broadly consistent with georegions used for the phylogeographic model. The quantity of exports and imports was compared for each region and only the maximum trade quantity linking two regions was recorded due to inconsistencies in data reported.

### Distribution of isolates and dataset design

All available H9 influenza A HA gene sequences were downloaded from the Influenza Virus Resource database (http://www.ncbi.nlm.nih.gov/genomes/FLU/FLU.html) on March 30, 2014. Accession numbers of newly sequenced viruses are presented in S1 Table. The data analyzed, including isolation dates, latitude, longitude and accession numbers are presented in the attached S1 Dataset. Sequences included in the dataset were subject to the following criteria: a) sequences had known location, host, and isolation date; b) for sequences with the same location, date of isolation and 100% similarity a single representative was retained; c) vaccine, derivative, and recombinant sequences were excluded; and d) sequences less than 1000 nucleotides in length were excluded. Locations with fewer than 10 taxa, as well as taxa collected prior to 1970 were excluded.

The remaining taxa were coded by both geographic region and ecosystem (‘wild’ or ‘domestic’). The wild classification included migratory birds (i.e. *Anseriformes*, *Charadriiformes*, etc.). The agricultural ecosystem included domestic birds raised for consumption (*Galliformes* including chicken, quail and pheasant; and *Anseriformes* including domestic duck and goose). See S1 Text for detailed descriptions of data stratification and subsampling. The final dataset consisted of 955 taxa, which were coded into 9 geographic regions: Japan/South Korea (n = 116), China–East (n = 147), China–Central (n = 179), China–West (n = 94), Southeast Asia (n = 18), South Asia (n = 93), Middle East (n = 210), Europe (n = 36), and North America (n = 62). 178 taxa were isolated from wild birds and the remaining from domesticated poultry. S2 Table presents detailed stratification of the dataset by region and ecosystem and S2 Fig shows the H9-HA sequence/location/year before and after subsampling.

Two additional datasets were assembled to investigate if model inferences from analysis of the H9 dataset could be generalized to other influenza A virus subtypes. Low pathogenic avian influenza A H3 and H6 subtype viruses have been sampled from both ecosystems and were chosen as comparison datasets. Even though AIV has a global distribution, surveillance and reporting is inconsistent and data availability for both wild and domestic birds can be limited. The AIV H3 and H6 subtype spatial distribution of wild and domestic birds sampled were similar to those of H9, but not identical. For the H3 subtype, sequence data was available from North Asia, including Russia and Mongolia, but none were available from western China, South Asia or the Middle East. For the H6 subtype no sequence data was available from South Asia. Limiting our ancestral state reconstruction to location states that overlapped between datasets would result in the exclusion of substantial data. Variation in data availability, locations sampled, and dataset design is discussed in the S1 Text. All available H3 and H6 subtype HA gene sequence data was downloaded from the Influenza Virus Resource database and screened based on the criteria described above (S2 Table).

### Bayesian phylogenetic and coalescent analysis

For each of the gene segments analyzed, Bayesian phylogenetic trees were estimated using BEAST v.1.8 [43] with an uncorrelated lognormal relaxed molecular clock [44] that allows for rate variation across lineages. The general time-reversible model of nucleotide substitution (+ gamma + invariant sites) was used along with a Bayesian skyline coalescent tree prior. A minimum of three independent runs of 150 million generations were performed and combined after removal of burn-in to achieve an Effective Sample Size of >200 as diagnosed in Tracer v1.6.

### Ancestral state reconstruction of host ecology and location of isolation

Phylogenies record the history of viral exchange between ecosystems and the gene flow between spatially sampled populations [4, 6, 7, 32, 45–48]. By integrating both ecosystem and geography into the phylogenetic model, we can estimate the relative contribution of each to the global distribution and diversity of viruses in circulation. We used a non-reversible continuous-time Markov chain model to estimate the migration rates between geographical regions and the general patterns of H9 virus circulation in different avian populations [25]. Here we estimate the network linking the discrete wild and domestic populations distributed across regions. By defining the geographically and ecologically discrete characters in our model we were able to distinguish whether inter-regional migration was between domestic populations or wild animals. In addition, we estimated the rate of viral transmission between wild and domestic flocks and where the ecosystem interface was porous. A limitation of this approach is that realistic measurements of bird density and disease prevalence were not accounted for within our model.

A Bayesian stochastic search variable selection (BSSVS) was employed to reduce the number of parameters to those with significantly non-zero transition rates [25]. The BSSVS explores and efficiently reduces the state space by employing a binary indicator (*I*). A Bayes factor (BF) can be computed to assess the support for individual transitions between discrete states. We identify a transition as important when P(I = 1|data) >0.5. This analysis was conducted with a Poisson prior on the number of non-zero rates with a mean equal to the minimum number of rates required to connect the discrete ecological/geographical region states. We applied this to our analysis of the H9 dataset and determined the critical BF >14 (Poisson prior mean = 15). The same criterion was applied to our analysis of H3 (Poisson prior mean = 11) and H6 (Poisson prior mean = 14) datasets and determined a critical Bayes factor >10 and > 12 respectively. Strength of statistical supports were interpreted as follows; 10≤ BF <30 indicating strong support, 30≤ BF <100 indicates very strong support and BF >100 indicating decisive support [8, 25, 32].

We assessed statistical support of rate differences (wild > domestic and domestic < wild) by computing Bayes factors. The Bayes factors for differences in migration rates (*r*) were estimated by the ratio of posterior odds (P(*r*_1_ > *r*_2_ | Data)/P(*r*_2_ > *r*_1_ | Data)) versus prior odds P(*r*_1_ > *r*_2_)/P(*r*_2_ > *r*_1_), where the prior odds ratio was approximately 1 [8, 32].

### Model sensitivity to sampling bias

Domestic populations were sampled much more intensively than wild populations (S1 Text). To investigate if our reconstruction was sensitive to data heterogeneity, we consider the prior expectation for the root discrete state frequencies and mean ecosystem transition rates for the sampling scheme used. If the discrete state distribution at the root is correlated with the location frequencies at the tips, we can expect that ancestral reconstruction throughout the entire phylogeny will be influenced by this tip-location sampling frequency. We randomized the location assignments at the tips throughout the MCMC procedure to investigate this possibility [37]. Similarly, if uneven sampling influences ecosystem transition rates then we can expect the mean rates to deviate from the prior. We further tested the model sensitivity to alternative sampling procedures where the number of sequences sampled was randomly pruned from a maximum of 955 sequences to a minimum of 73 sequences. Analysis of each sampling scheme was repeated three times. This sensitivity analysis was carried out for the final H3 and H6 subtype datasets. Our results quickly converged on the prior root location probability for all subtypes (equal state frequencies; S5 Fig) and ecosystem transition rate probability (equal mean rates; S6 Fig) indicating that the sampling frequencies had little impact on the model inferences *a posteriori*.

### Source/sink dynamics and relative risk of viral emergence

To assess the contribution of each region as a viral source or sink in the migration network, state jumps at the tree nodes, representing a state transition event (i.e. migration or ecosystem interaction) were counted [36]. We used a non-reversible model and therefore the direction of gene flow between states can be determined to assess which region was either source or sink. Heat maps representing the average number of jumps per year estimated from the last 1000 posterior sampled trees were generated. Due to the increase in surveillance efforts post-1997’s Hong Kong H5N1 outbreak and recent intensification in poultry production, our analysis only considered migration events between 1998 and 2013.

Even though the explicit migration events are not observed, the waiting times between state changes can be tracked on the phylogeny. The duration that a particular state is observed before transitioning to another state (Markov reward) was recorded on a branch-by-branch basis from the posterior sampling of phylogenetic trees [49]. These Markov rewards were calculated for each tree and ecosystem/location state in our model.

We further assessed the relative risk of each location as a viral source or sink population using 2x2 contingency tables [50]. The total jump counts in to or out of each discrete state were obtained for each step of the Bayesian MCMC. Contingency tables containing four cells (A, B, C, and D) were populated for each combination of regions. For example, to calculate the relative proportion of times viruses emerged from Japan and migrated to North America, the columns of the contingency table denote events in which Japan was the geographic source (left column) and events in which Japan was not the source (right column). Likewise, the rows of the contingency table can be denoted as events in which North America was the geographic sink of a transition (top row) and events in which was not the sink (bottom row). Therefore, cell A in this example includes the total number of estimated events in which viruses from Japan were introduced into North America; cell B includes the total introductions from other regions (not Japan) into North America; cell C includes the total number of introductions from Japan into other regions (not North America), and cell D includes the total number of mutually exclusive events in which Japan was not the geographic source and North America was not the geographic sink. The relative proportion of introductions from Japan into North America will be calculated as [A/(A+B)]/[C/(C+D)]. The proportion of total times where North America was the sink when Japan was the source was represented by [A/(A+B)]. Likewise, [C/(C+D)] represents the proportion of total times when viruses entered other regions (not North America) where Japan was the source. The ratio of these proportions represent the relative risk of Japan as the source when North America was the sink compared to when other regions were the geographic sinks. These ratios were calculated per MCMC step and averaged across all steps for each in order to incorporate phylogenetic uncertainty.

Data deposited in the Dryad repository: http://dx.doi.org/10.5061/dryad.601fd. [51]

### Ethics statement

All studies involving the collection of samples from wild and domestic animal species are conducted in compliance with the policies of the National Institutes of Health and the Animal Welfare Act, and with the approval of the St. Jude Children's Research Hospital Institutional Animal Care and Use Committee (Protocol Number 546-100324-10/14, approved July 20, 2015) and Massachusetts Institute for Technology (Protocol Number 0515-046-18, approved May 3, 2015).

## Supporting Information

S1 Text Dataset descriptions and model extensions. This file contains details of the sampling procedures used and descriptions of the final datasets presented in this manuscript.
(PDF)

S1 Table Accession numbers of newly sequenced viruses. (PDF)

S2 Table Dataset summary before and after subsampling. (PDF)

S3 Table Mean migration rates of H3 subtype viruses between avian ecosystems across sampling locations. Statistically supported interactions are shown in bold.
(PDF)

S4 Table Mean migration rates of H6 subtype viruses between avian ecosystems across sampling locations. Statistically supported interactions are shown in bold.
(PDF)

S1 Dataset Ecotype, Latitude and Longitude of globally distributed samples (XLSX)

S1 Fig Map showing global duck production density. (PDF)

S2 Fig **(A)** Histogram of H9 avian influenza isolates included in the full dataset per year by regions. **(B)** Histogram of H9 avian influenza isolates following subsampling included in the final dataset per year by regions.
(PDF)

S3 Fig Bayesian relaxed clock phylogenetic MCC tree of global H9 HA gene sequences with taxon names. Purple bars on nodes indicate 95% Bayesian credibility intervals of divergence time estimates.
(PDF)

S4 Fig Heat map showing source-sink dynamics/location/year. (PDF)

S5 Fig Inferred migration rates and patterns for H3 subtype viruses sampled from wild and domestic animals. **(A)** Map showing statistically supported transitions between geographic regions by ecosystem. Line thickness corresponds to viral flow rates shown in S3 Table (thinnest <0.5; 0.5 to <1; 1 to <2; ≥2 thickest). (**B)** Density distribution of statistically supported mean transition rates between ecosystems. (**C)** Statistically supported mean migration rates per MCMC step of wild-to-wild avian transitions versus domestic-to-domestic avian transitions.
(PDF)

S6 Fig Bayesian relaxed clock phylogenetic MCC tree of global H3 HA gene sequences with taxon names. Purple bars on nodes indicate 95% Bayesian credibility intervals of divergence time estimates.
(PDF)

S7 Fig Inferred migration rates and patterns for H6 subtype viruses sampled from wild and domestic animals. **(A)** Map showing statistically supported transitions between geographic regions by ecosystem. Line thickness corresponds to viral flow rates shown in S4 Table (thinnest <0.5; 0.5 to <1; 1 to <2; ≥2 thickest). (**B)** Density distribution of statistically supported mean transition rates between ecosystems. (**C)** Statistically supported mean migration rates per MCMC step of wild-to-wild avian transitions versus domestic-to-domestic avian transitions.
(PDF)

S8 Fig Bayesian relaxed clock phylogenetic MCC tree of global H6 HA gene sequences with taxon names. Purple bars on nodes indicate 95% Bayesian credibility intervals of divergence time estimates.
(PDF)

S9 Fig Root state probabilities as a function of number of taxa sampled from a particular state estimated during tip-state randomization procedure. The final dataset is shown in green and alternative state sampling procedures indicated in blue. Grey line indicates the prior expectation for the root location probability. Shaded area indicates empirical posterior probability under conditions of over-parametization (i.e. more parameters estimated than data points observed).
(PDF)

S10 Fig Mean state transition rates within and between ecosystems using tip-state randomization for the full dataset and alternative state sampling procedures (A-G). The number of sequenced used in each analysis were A) n = 73; B) n = 143; C) n = 260; D) n = 424; E) n = 564; F) n = 686; G) n = 791.
(PDF)

S11 Fig A) Mean state transition rates within and between ecosystems and B) root state probability using tip-state randomization for the H3 dataset. (PDF)

S12 Fig A) Mean state transition rates within and between ecosystems and B) root state probability using tip-state randomization for the H6 dataset. (PDF)
