# Supplementary material for: Ecosystem Interactions Underlie the Spread of Avian Influenza A Viruses with Pandemic Potential
Source: PLoS Pathog. 2016 May 11;12(5):e1005620. doi: 10.1371/journal.ppat.1005620 (PMC4864295; doi:10.1371/journal.ppat.1005620)
Supplement: S1 Text — This file contains details of the sampling procedures used and descriptions of the final datasets presented in this manuscript. (PDF) [file ppat.1005620.s001.pdf]

Ecosystem interactions underlie the spread of avian influenza A viruses with pandemic potential

Justin Bahl[1,2]*, Truc T. Pham[1], Nichola J. Hill[3], Islam T. M. Hussein[3], Eric J. Ma[3], Bernard C. Easterday[4], Rebecca A. Halpin[5], Timothy B. Stockwell[5], David E. Wentworth[5], Ghazi Kayali[6], Scott Krauss[6], Stacey Schultz-Cherry[6], Robert G. Webster[6], Richard J. Webby[6], Michael D. Swartz[1], Gavin J.D. Smith[2,7], Jonathan A. Runstadler[3]*

Short title: Influenza transmission between wild and domestic populations

Affiliations:

[1]Center for Infectious Diseases, The University of Texas School of Public Health, Houston, Texas, United States of America

[2]Program in Emerging Infectious Diseases, Duke-National University of Singapore Graduate Medical School, 8 College Road, Singapore 169857, Singapore

[3]Division of Comparative Medicine, Department of Biological Engineering, Massachusetts Institute of Technology, Cambridge, Massachusetts, USA

[4]Department of Pathobiological Sciences, School of Veterinary Medicine, University of Wisconsin-Madison

[5]J. Craig Venter Institute, Rockville, Maryland, United States of America

[6]Department of Infectious Diseases, St. Jude Children's Research Hospital, Memphis, Tennessee, United States of America

[7]Duke Global Health Institute, Duke University, Durham, North Carolina, USA.

*Corresponding authors

E-mail: Justin.Bahl@uth.tmc.edu or jrun@mit.edu

Dataset Descriptions

Experimental Design

H9 subtype viruses are commonly isolated from domestic poultry and wild birds (3,9,18). Periodic human infections have resulted in intensive surveillance of domestic birds in order to identify the sources of infection (16,17). As a result, a robust dataset of H9 subtype viruses is available from both domestic and wild birds throughout the world. Since poultry and wild birds infected with H9 viruses do not often manifest major disease symptoms, vaccination or active control efforts are limited for this subtype. We utilize the robust H9 virus surveillance and sequence reporting, enhanced with novel sequences acquired from North American wild bird surveillance collected between 1974 and 2013. Accession numbers of newly sequenced viruses are presented in Table S1. The data analyzed, including isolation dates, latitude, longitude and accession numbers are presented in Supplementary Data File 2.

Distribution of Isolates and Dataset Design

All available H9 influenza A HA gene sequences were downloaded from the Influenza Virus Resource database (http://www.ncbi.nlm.nih.gov/genomes/FLU/FLU.html) on March 30, 2014. Sequences in the dataset were subject to the following criteria: a) sequences had known location, host, and isolation date; b) for sequences with the same location, date of isolation and 100% similarity a single representative was retained; c) vaccine, derivative, and recombinant sequences were excluded; and d) sequences less than 1000 nucleotides in length were excluded. For this study, only sequences collected from avian hosts were included; sequences collected from the environment were retained as well since these often come from avian fecal samples (26). This dataset consisted of 2341 taxa. Taxa from South America (n=1), Australia (n=7), New Zealand (n=1), Russia (n=2), Mongolia (n=1), and Africa (n=3) were excluded due to small

sample sizes. Taxa collected prior to 1976 (n=8) were excluded to guard against storage or sequencing artefacts. Eighteen taxa were also excluded due to poor sequence quality.

The remaining taxa were coded by both geographic region and ecosystem (‘wild’ or ‘domestic’) in order to assess how these factors influenced the H9 viral population structure. Virus sampling sites were grouped into nine discrete geographic states based on the global distribution of samples (Figure 1). These regions included Japan/South Korea (n=116), China – East (n=583), China – Central (n=854), China – West (n=129), Southeast Asia (n=18), South Asia (n=93), Middle East (n=347), Europe (n=36) and North America (n=107). Each region consisted of several adjacent countries or administrative units that constituted a unique agro-ecological system (Figure 1). Due to its expansive geographic area, Mainland China was subdivided into three regions based on sample distribution and intensity of poultry production (Figure 1C).

The wild classification included migratory birds (i.e. *Anseriformes*, *Charadriiformes*, etc.). Wild birds used for recreation, including falconry (i.e. bustards and falcons), were excluded from the analysis. The agricultural ecosystem classification included domestic poultry raised for consumption (*Galliformes* including chicken, quail and pheasant; and *Anseriformes* including domestic duck and goose) (Supplementary Data). Seventeen taxa did not have sufficient species information available to code the ecosystem and were therefore excluded. Many isolates in GenBank identify the host species as “duck.” For each isolate where limited information was available, we 1) determined if any published reference presented additional information on the host species/ecosystem; 2) assumed the sample was domestic if there was no history of wild bird surveillance in the region; 3) excluded the data from the analysis if the assignment to either ecosystem character was ambiguous.

Stratification of this full dataset by ecosystem and geographical region revealed that taxa coded into the domestic category comprised the majority of isolates, particularly from the Middle East and China. To reduce sampling biases, we randomly subsampled each region per year (Figure S2). The number of sequences available in the public database from domestic poultry increased after 2000. Therefore, sequences collected prior to this period were randomly subsampled to retain at least 10 sequences per geographic region per year. Regions that originally had less than 10 domestic poultry sequences prior to 2000 were not subsampled. Sequences collected post-2000 were stratified by both country (or for China by province) and year of isolation to account for increased sampling when outbreaks occurred. For regions with large numbers of domestic samples, 10 sequences from each year and region were randomly subsampled and retained for the final dataset. Due to the smaller number of wild bird sequences available, all these sequences were retained from every region with the exception of North America. Examination of this dataset revealed a large number of wild bird sequences collected from Delaware Bay in 2003, and isolates collected from this region had low genetic diversity indicating a localized outbreak. We therefore subsampled data from this location by half. This subsampling procedure was repeated three times and estimated migration parameters were averaged from independent Bayesian simulations of each subsampled dataset.

The final dataset used for analysis consisted of 955 taxa, which were coded into nine geographic regions: Japan/South Korea (n=116), China – East (n=147), China – Central (n=179), China – West (n=94), Southeast Asia (n=18), South Asia (n=93), Middle East (n=210), Europe (n=36), and North America (n=62). 178 taxa were isolated from wild birds and the remaining from domesticated poultry. Table S2 presents detailed stratification of the dataset by region and ecosystem.

Extensions to other HA subtypes

To extend our analyses to other AIV subtypes, all available H3 and H6 influenza A HA gene sequences were downloaded from the Influenza Virus Resource database on November 10, 2015 and subjected to the same inclusion and exclusion processes as the H9 dataset. 1490 taxa were available for AIV H3 viruses, of which 106 taxa were further excluded due to sparse sampling locations (n=30), collections prior to 1976 (n=23), missing species information (n=29), or poor sequence quality (n=24). Based on the global distribution of these H3 virus samples, collection sites were grouped into seven discrete geographic states (Table S2): Japan/South Korea, China – East, China – Central, Southeast Asia, Europe, North America, and North Asia which was warranted by the presence of wild bird isolates collected from Russia and Mongolia. The Middle East, South Asia, and western China were not were represented.

In contrast to the data available for H9 subtype, stratification of this dataset by both ecosystem and geographical region revealed that AIV H3 sequences were more heavily sampled among wild birds than domestic poultry. North America, particularly the United States, comprised the majority of the wild bird H3 viruses (Table S2). Therefore sequences collected from this region prior to 2000 were randomly subsampled to retain at least 10 sequences per year. Sequences collected post-2000 were stratified by both state and year of isolation to account for increased sampling and/or outbreaks that occurred. They were then randomly subsampled to retain 10 sequences from each year and region for the final dataset. The majority of H3 viruses from domestic poultry were retained with the exception of a relatively high number of genetically similar duck sequences from South Korea in 2007, and as a result were subsampled. The final H3 subtype dataset consisted of 814 taxa.

Analysis of H3 subtype HA gene sequences showed similar results to those described above for H9 (Fig. S7, S8, Table S3). H3 virus migration in East, Central and South East Asia was dominated by transitions between domestic populations (Fig. S7). However, the importance of these transitions to the global distribution and persistence of H3 is unclear. The majority of H3 HA sequences in the global dataset were isolated from North American wild birds in which the virus circulates endemically (Table S1). Despite uncertainty in reporting, viral transmission between wild and domestic systems play an important role in shaping H3 influenza A virus population structure as evidenced from the phylogenetic tree (Fig. S8) where wild populations dominate the tree backbone, in contrast to observations from the H9 subtype analysis.

The role of virus ecosystem interactions in determining viral distribution of H6 subtype viruses was less clear (Fig. S8). Of the 1560 taxa available for AIV H6 viruses, 78 taxa were further excluded due to sparse sampling locations (n=28), collections prior to 1976 (n=38), missing species information (n=6), or poor sequence quality (n=6). Preliminary phylogenetic analyses of the data suggested that a monphyletic group (n=135) consisting of samples from the Midwest United States was genetically distinct from other sequences and was removed from the final dataset. Based on the global distribution of the remaining H6 virus samples, collection sites were grouped into seven discrete geographic states (Table S2): Japan/South Korea, China – East, China – Central, China – West, Southeast Asia (n=18), Middle East, Europe and North America.

Although stratification of this dataset by ecosystem revealed that the number of AIV H6 sequences in the dataset were comparable, viruses from wild birds were largely collected from North America and Europe whereas isolates in domestic poultry were primarily from Asia (Table S2). Further characterization of the data by collection year indicated an increase in the number of domestic poultry H6 isolates following 2000 from China. North America and Europe also had

increased H6 virus isolations following 2004. These ecotypes and regions were therefore randomly subsampled during these time periods to retain 10 sequences from each year and region for the final H6 subtype dataset (N=888 taxa).

Despite evidence of frequent two-way transmission between wild or domestic populations (Fig. S8, S9 Table S4), our results show no support for either ecosystem playing a larger role in the distribution of these viruses. Transitions into chickens appear to be dead-end transmissions (Fig. S9). It remains unclear if our results were an artefact of the binning procedure used to assign discrete states or if another biological process (i.e. host bird preference or transmission mechanism) might play a stronger role in the spread of H6 viruses. Even though interactions between ecosystems likely perpetuate the spread of H6 viruses, more granular sampling strategies that integrate parameters such as city or state, host species and population prevalence are needed to investigate underlying processes determining risk of H6 virus spread.
