# Supplementary material for: Ecosystem Interactions Underlie the Spread of Avian Influenza A Viruses with Pandemic Potential
Source: PLoS Pathog. 2016 May 11;12(5):e1005620. doi: 10.1371/journal.ppat.1005620 (PMC4864295; doi:10.1371/journal.ppat.1005620)
Supplement: S1 Table — (PDF) [file ppat.1005620.s002.pdf]

Table S1: Accession numbers of newly sequenced viruses

Strain name	PB2	PB1	PA	HA	NP	NA	M	NS
A/ruddy turnstone/Delaware Bay/215/1996(H9)	CY101222	CY101221	CY101219	CY101211	CY101215	CY101214	CY101213	CY101217
A/shorebird/Delaware Bay/260/1996(H9N9)	CY101231	CY101230	CY101229	CY101224	CY101227	CY101226	CY101225	CY101228
A/shorebird/Delaware Bay/133/2002(H9N1)	CY101322	CY101321	CY101320	CY101315	CY101318	CY101317	CY101316	CY101319
A/shorebird/Delaware Bay/73/2003(H9N2)	CY101329		CY101328	CY101323	CY101326	CY101325	CY101324	CY101327
A/laughing gull/Delaware Bay/2718/1987(H9N5)	CY101397	CY101396	CY101395	CY101390	CY101393	CY101392	CY101391	CY101394
A/ruddy turnstone/Delaware Bay/2774/1987(H9N5)	CY101405	CY101404	CY101403	CY101398	CY101401	CY101400	CY101399	CY101402
A/ruddy turnstone/Delaware Bay/2795/1987(H9N5)	CY101413	CY101412	CY101411	CY101406	CY101409	CY101408	CY101407	CY101410
A/ruddy turnstone/Delaware Bay/2830/1987(H9N5)	CY101421	CY101420	CY101419	CY101414	CY101417	CY101416	CY101415	CY101418
A/ruddy turnstone/Virginia/2297/1988(H9N9)	CY101514	CY101513	CY101512	CY101507	CY101510	CY101509	CY101508	CY101511
A/laughing gull/Delaware/2971/1988(H9N9)	CY101546	CY101545	CY101544	CY101539	CY101542	CY101541	CY101540	CY101543
A/shorebird/Delaware Bay/31/1996(H9N7)	CY102195	CY102194	CY102193	CY102188	CY102191	CY102190	CY102189	CY102192
A/ruddy turnstone/Delaware Bay/261/1999(H9N7)	CY102539	CY102538	CY102537	CY102532	CY102535	CY102534	CY102533	CY102536
A/shorebird/Delaware Bay/277/2000(H9N7)	CY102640	CY102639	CY102638	CY102633	CY102636	CY102635	CY102634	CY102637
A/laughing gull/Delaware Bay/5/2003(H9N1)	CY102727	CY102726	CY102725	CY102720	CY102723	CY102722	CY102721	CY102724
A/shorebird/Delaware Bay/127/2003(H9N2)	CY102735	CY102734	CY102733	CY102728	CY102731	CY102730	CY102729	CY102732
A/shorebird/Delaware Bay/246/2003(H9N5)	CY102743	CY102742	CY102741	CY102736	CY102739	CY102738	CY102737	CY102740
A/shorebird/Delaware Bay/283/2003(H9N1)	CY102751	CY102750	CY102749	CY102744	CY102747	CY102746	CY102745	CY102748
A/sanderling/Delaware Bay/449/2006(H9N2)	CY103006	CY103005	CY103004	CY102999	CY103002	CY103001	CY103000	CY103003
A/ruddy turnstone/Delaware Bay/2813/1987(H9 mixed)	CY136367	CY136366	CY136365	CY136358	CY136363	CY136362	CY136359	CY136364
A/northern shoveler/Interior Alaska/8BM3470/2008 (H9N2)	CY079653	CY079652	CY079651	CY079646	CY079649	CY079648	CY079647	CY079650
A/American green-winged teal/Interior Alaska/ 10BM16586R0/2010(H9 mixed)	CY125741	CY125740	CY125739	CY125733	CY125737	CY125735	CY125734	CY125738
A/northern pintail/Interior Alaska/ 10BM14807R2/2010(H9N2)	CY125757	CY125756	CY125755	CY125750	CY125753	CY125752	CY125751	CY125754
A/northern shoveler/Interior Alaska/ 10BM16764R0/2010(H9N2)	CY125765	CY125764	CY125763	CY125758	CY125761	CY125760	CY125759	CY125762
A/ruddy turnstone/Delaware Bay/201/1996(H9 mixed)	CY126491	CY126490	CY126489	CY126482	CY126486	CY126485	CY126484	CY126487
A/mallard/Interior Alaska/10BM02980R0/2010(H9N2)	CY130460	CY130459	CY130458	CY130453	CY130456	CY130455	CY130454	CY130457
A/green-winged teal/Wisconsin/228/1976 (H9N2)	CY180146	CY180145	CY180144	CY180139	CY180142	CY180141	CY180140	CY180143
A/green-winged teal/Wisconsin/562/1979 (H9N1)	CY180715	CY180714	CY180713	CY180708	CY180711	CY180710	CY180709	CY180712
A/mallard/Wisconsin/24/1974 (H9N1)	CY181344	CY181343	CY181342	CY181337	CY181340	CY181339	CY181338	CY181341

A/blue-winged teal/Alberta/142/1992(H6N8)	CY126975	CY126974	CY126973	CY126968	CY126971	CY126970	CY126969	CY126972
A/blue-winged teal/Alberta/599/1986(H6N2)	CY126807	CY126806	CY126805	CY126800	CY126803	CY126802	CY126801	CY126804
A/green-winged teal/Alberta/107/1992(H6N8)	CY126967	CY126966	CY126965	CY126960	CY126963	CY126962	CY126961	CY126964
A/gull/Delaware Bay/18/2000(H6N1)	CY127621	CY127620	CY127619	CY127614	CY127617	CY127616	CY127615	CY127618
A/herring gull/Delaware Bay/77/1994(H6N8)	CY127055	CY127054	CY127053	CY127048	CY127051	CY127050	CY127049	CY127052
A/herring gull/New Jersey/413/1989(H6N3)	CY126767	CY126766	CY126765	CY126760	CY126763	CY126762	CY126761	CY126764
A/laughing gull/Delaware Bay/208/1994(H6N3)	CY127071	CY127070	CY127069	CY127064	CY127067	CY127066	CY127065	CY127068
A/laughing gull/Delaware Bay/261/1991(H6N8)	CY126935	CY126934	CY126933	CY126928	CY126931	CY126930	CY126929	CY126932
A/laughing gull/Delaware Bay/4/1990(H6N8)	CY126879	CY126878	CY126877	CY126872	CY126875	CY126874	CY126873	CY126876
A/laughing gull/New Jersey/177/1990(H6N8)	CY126887	CY126886	CY126885	CY126880	CY126883	CY126882	CY126841	CY126884
A/mallard/Alberta/110/1990(H6 mixed)	CY136417, CY136418	CY136416	CY136415	CY136406	CY136412	CY136410, CY136411	CY136408, CY136409	CY136413, CY136414
A/mallard/Alberta/152/1992(H6N8)	CY126991	CY126990	CY126989	CY126984	CY126987	CY126986	CY126985	CY126988
A/mallard/Alberta/174/1989(H6N8)	CY126847	CY126846	CY126845	CY126840	CY126843	CY126842	CY126841	CY126844
A/mallard/Alberta/186/1987(H6N2)	CY136661	CY136660	CY136659	CY136654	CY136657	CY136656	CY136655	CY136658
A/mallard/Alberta/19/1994(H6N8)	CY127095	CY127094	CY127093	CY127088	CY127091	CY127090	CY127089	CY127092
A/mallard/Alberta/195/1987(H6N2)	CY136669	CY136668	CY136667	CY136662	CY136665	CY136664	CY136663	CY136666
A/mallard/Alberta/196/1987(H6N2)	CY136677	CY136676	CY136675	CY136670	CY136673	CY136672	CY136671	CY136674
A/mallard/Alberta/196/1989(H6N8)	CY126458	CY126457	CY126456	CY126451	CY126454	CY126453	CY126452	CY126455
A/mallard/Alberta/197/1987(H6N2)	CY136685	CY136684	CY136683	CY136678	CY136681	CY136680	CY136679	CY136682
A/mallard/Alberta/203/1992(H6N5)	CY126999	CY126998	CY126997	CY126992	CY126995	CY126994	CY126993	CY126996
A/mallard/Alberta/207/1990(H6N3)	CY126903	CY126902	CY126901	CY126896	CY126899	CY126898	CY126897	CY126900
A/mallard/Alberta/215/1999(H6 mixed)	CY127613	CY127612	CY127610, CY127611	CY127604	CY127608	CY127606, CY127607	CY127605	CY127609
A/mallard/Alberta/232/1994(H6N8)	CY127127	CY127126	CY127125	CY127120	CY127123	CY127122	CY127121	CY127124
A/mallard/Alberta/250/1990(H6N3)	CY126911	CY126910	CY126909	CY126904	CY126907	CY126906	CY126905	CY126908
A/mallard/Alberta/257/1990(H6N3)	CY126919	CY126918	CY126917	CY126912	CY126915	CY126914	CY126913	CY126916
A/mallard/Alberta/260/1987(H6N2)	CY136709	CY136708	CY136707	CY136702	CY136705	CY136704	CY136703	CY136706
A/mallard/Alberta/264/1986(H6N2)	CY126360	CY126359	CY126358	CY126354		CY126356	CY126355	CY126357
A/mallard/Alberta/270/1986(H6N2)	CY126775	CY126774	CY126773	CY126768	CY126771	CY126770	CY126769	CY126772
A/mallard/Alberta/294/1987(H6 mixed)	CY136728	CY136727	CY136726	CY136719	CY136723	CY136722	CY136720, CY136721	CY136724, CY136725
A/mallard/Alberta/311/1986(H6N2)	CY126368	CY126367	CY126366	CY126361	CY126364	CY126363	CY126362	CY126365
A/mallard/Alberta/4/1994(H6N8)	CY127079	CY127078	CY127077	CY127072	CY127075	CY127074	CY127073	CY127076
A/mallard/Alberta/57/1994(H6N8)	CY126466	CY126465	CY126464	CY126459	CY126462	CY126461	CY126460	CY126463
A/mallard/Alberta/58/1989(H6N4)	CY126449, CY126450	CY126447, CY126448	CY126446	CY126441	CY126444	CY126443	CY126442	CY126445
A/mallard/Alberta/68/1994(H6N8)	CY127111	CY127110	CY127109	CY127104	CY127107	CY127106	CY127105	CY127108
A/pintail/Alberta/144/1989(H6N8)	CY126815	CY126814	CY126813	CY126808	CY126811	CY126810	CY126809	CY126812
A/pintail/Alberta/145/1990(H6N3)	CY136760	CY136759	CY136758	CY136753	CY136756	CY136755	CY136754	CY136757
A/pintail/Alberta/155/1994(H6N8)	CY131983	CY131982	CY131981	CY131976	CY131979	CY131978	CY131977	CY131980
A/pintail/Alberta/267/1987(H6N2)	CY136717	CY136716	CY136715	CY136710	CY136713	CY136712	CY136711	CY136714
A/pintail/Alberta/314/1986(H6N2)	CY126383	CY126382	CY126381	CY126376	CY126379	CY126378	CY126377	CY126380
A/pintail/Alberta/480/1986(H6N2)	CY126411, CY126412	CY126410	CY126409	CY126404	CY126407	CY126406	CY126405	CY126408
A/pintail/Alberta/87/1993(H6N8)	CY127031	CY127030	CY127029	CY127024	CY127027	CY127026	CY127025	CY127028
A/redhead/Alberta/291/1994(H6N8)	CY127135	CY127134	CY127133	CY127128	CY127131	CY127130	CY127129	CY127132
A/ruddy turnstone/Delaware Bay/113/1998(H6N8)	CY127428	CY127427	CY127426	CY127421	CY127424	CY127423	CY127422	CY127425
A/ruddy turnstone/Delaware Bay/118/2007(H6 mixed)	CY127782	CY127781	CY127780	CY127774	CY127778	CY127777	CY127776	CY127779
A/ruddy turnstone/Delaware Bay/125/1998(H6 mixed)	CY126502, CY126503	CY126501	CY126499, CY126500	CY126493	CY126497	CY126495, CY126496	CY126494	CY126498
A/ruddy turnstone/Delaware Bay/126/1994(H6N1)	CY136789	CY136788	CY136787	CY136782	CY136785	CY136784	CY136783	CY136786
A/ruddy turnstone/Delaware Bay/136/2007(H6N1)	CY127790	CY127789	CY127788	CY127783	CY127786	CY127785	CY127784	CY127787
A/ruddy turnstone/Delaware Bay/162/1994(H6N2)	CY127063	CY127062	CY127061	CY127056	CY127059	CY127058	CY127057	CY127060
A/ruddy turnstone/Delaware Bay/173/2007(H6N2)	CY127798	CY127797	CY127796	CY127791	CY127794	CY127793	CY127792	CY127795
A/ruddy turnstone/Delaware Bay/190/2002(H6 mixed)	CY126554	CY126553	CY126552	CY126546	CY126550	CY126548, CY126549	CY126547	CY126551
A/ruddy turnstone/Delaware Bay/203/1996(H6N2)	CY127396	CY127395	CY127394	CY127389	CY127392	CY127391	CY127390	CY127393
A/ruddy turnstone/Delaware Bay/255/1991(H6N2)	CY126927	CY126926	CY126925	CY126920	CY126923	CY126922	CY126921	CY126924
