# Supplementary material for: Ecosystem Interactions Underlie the Spread of Avian Influenza A Viruses with Pandemic Potential
Source: PLoS Pathog. 2016 May 11;12(5):e1005620. doi: 10.1371/journal.ppat.1005620 (PMC4864295; doi:10.1371/journal.ppat.1005620)
Supplement: S2 Table — (PDF) [file ppat.1005620.s003.pdf]

Table S2. Dataset summary before and after subsampling.

H9 AIV Sequences

	Japan/ S Korea	E China	C China	W China	SE Asia	S Asia	Middle East	Europe	N America	Total
Domestic	100	570	816	129	17	91	326	11	9	2069
Wild	16	13	38	0	1	2	21	25	98	214
Region total	116	583	854	129	18	93	347	36	107	**2283**
After subsampling										
Domestic	100	134	141	94	17	91	189	11	0	777
Wild	16	13	38	0	1	2	21	25	62	178
Region total	116	147	179	94	18	93	210	36	62	**955**

H3 AIV Sequences

	North Asia	Japan/ S Korea	E China	C China	SE Asia	Europe	N America	Total
Domestic	0	62	23	31	24	0	3	143
Wild	46	52	2	8	8	86	1039	1241
Region total	46	114	25	39	32	86	1042	**1384**
After subsampling								
Domestic	0	46	23	31	24	0	3	**127**
Wild	46	52	2	8	8	63	508	687
Region total	46	98	25	39	32	63	511	**814**

H6 AIV Sequences

	Japan/ S Korea	E China	C China	W China	SE Asia	Middle East	Europe	N America	Total
Domestic	66	182	396	17	14	0	24	28	727
Wild	20	17	31	0	8	5	109	430	620
Region total	86	199	427	17	22	5	133	458	**1347**
After subsampling									
Domestic	66	87	202	17	14	0	22	25	433
Wild	18	17	31	0	8	5	88	288	455
Region total	84	104	233	17	22	5	110	313	**888**
