# Supplementary material for: Ecosystem Interactions Underlie the Spread of Avian Influenza A Viruses with Pandemic Potential
Source: PLoS Pathog. 2016 May 11;12(5):e1005620. doi: 10.1371/journal.ppat.1005620 (PMC4864295; doi:10.1371/journal.ppat.1005620)
Supplement: S3 Table — Statistically supported interactions are shown in bold. (PDF) [file ppat.1005620.s004.pdf]

Table S3. Mean migration rates of H3 subtype viruses between avian ecosystems across sampling locations. Statistically supported interactions are shown in bold.

		Destination											
		Domestic						Wild					
		Japan/ S Korea	E China	C China	SE Asia	North America	Russia/ Mongolia	Japan/ S Korea	E China	C China	SE Asia	Europe	North America
Origin: Domestic	Japan/ S Korea	-	0.36	0.31	0.32	0.36	0.27	**1.19§ (0.20, 2.46)**	0.39	0.31	0.29	0.33	0.33
	E China	0.53	-	0.73	**1.18‡ (0.03, 2.55)**	0.47	0.41	0.50	0.41	0.44	0.38	0.44	0.44
	C China	0.47	**3.46§ (1.36, 5.88)**	-	1.11	0.40	1.08	1.73	0.39	**0.99§ (0.10, 2.10)**	0.33	0.42	0.60
	SE Asia	0.40	0.44	**1.16§ (0.10, 2.59)**	-	0.48	0.38	0.41	0.45	0.40	**2.17§ (0.62, 3.95)**	0.35	0.40
	North America	0.37	0.36	0.36	0.37	-	0.35	0.63	0.40	0.36	0.38	0.40	0.40
	Russia/ Mongolia	0.47	0.70	**1.04‡ (0.01, 2.95)**	**2.18§ (0.70, 4.02)**	0.41	-	1.51	0.38	0.58	0.51	0.31	0.74
Origin: Wild	Japan/ S Korea	**0.59§ (0.04, 1.34)**	0.84	**2.19‡ (0.26, 4.10)**	**1.05§ (0.10, 2.19)**	0.42	**1.85‡ (0.48, 3.49)**	-	**0.41‡ (0.01, 1.03)**	0.54	0.24	0.71	**1.06§ (0.17, 2.14)**
	E China	0.35	0.38	0.39	0.41	0.48	0.38	0.38	-	0.36	0.37	0.41	0.40
	C China	0.39	0.38	0.42	0.40	0.44	0.37	0.40	0.42	-	0.37	0.39	0.41
	SE Asia	0.38	0.36	0.39	0.39	0.42	0.39	0.37	0.37	0.38	-	0.38	0.39
	Europe	0.22	0.24	0.67	0.19	0.24	**0.69‡ (0.04, 1.58)**	0.98	0.28	0.20	0.22	-	0.51
	North America	0.14	0.08	0.11	0.09	**0.29§ (0.04, 0.63)**	0.10	**0.19‡ (0.00, 0.47)**	0.10	0.09	0.10	0.17	-

BSSV statistically supported migration rates with 95% Bayesian credible intervals (BCI), where the Bayes factor was >10. ‡ 30 > BF ≥ 100; § BF > 100
