# Supplementary material for: Ecosystem Interactions Underlie the Spread of Avian Influenza A Viruses with Pandemic Potential
Source: PLoS Pathog. 2016 May 11;12(5):e1005620. doi: 10.1371/journal.ppat.1005620 (PMC4864295; doi:10.1371/journal.ppat.1005620)
Supplement: S4 Table — Statistically supported interactions are shown in bold. (PDF) [file ppat.1005620.s005.pdf]

Table S4. Mean migration rates of H6 subtype viruses between avian ecosystems across sampling locations. Statistically supported interactions are shown in bold.

Origin		Destination: Domestic Japan/ S Korea	Domestic E China	Domestic C China	Domestic W China	Domestic SE Asia	Domestic Europe	Domestic North America	Wild Japan/ S Korea	Wild E China	Wild C China	Wild SE Asia	Wild Middle East	Wild Europe	Wild North America
Domestic	Japan/ S Korea	-	0.33	0.32	0.19	0.19	0.15	0.15	0.68	0.29	0.23	0.24	0.26	0.29	0.20
Domestic	E China	0.68	-	1.26	0.33	1.10	0.26	0.17	**0.56[†] (0.01, 1.42)**	0.74	0.45	0.26	0.32	0.47	0.44
Domestic	C China	0.59	**2.42[§] (1.07, 4.01)**	-	**0.61[§] (0.08, 1.28)**	**0.65[†] (0.09, 1.29)**	0.17	0.09	0.56	**0.61[†] (0.02, 1.43)**	**2.55[§] (1.23, 3.94)**	0.21	0.14	0.31	0.59
Domestic	W China	0.42	0.36	0.49	-	0.41	0.29	0.39	0.32	0.47	0.35	0.61	0.42	0.37	0.42
Domestic	SE Asia	0.37	0.35	0.27	0.32	-	0.41	0.22	0.34	0.41	0.36	**1.68[§] (0.40, 3.26)**	0.28	0.25	0.32
Domestic	Europe	0.34	0.29	0.36	0.28	0.38	-	0.37	0.39	0.35	0.30	0.39	0.45	1.35	0.57
Domestic	North America	0.27	0.26	0.38	0.31	0.37	0.27	-	0.48	0.39	0.39	0.36	0.32	0.55	**0.60[§] (0.01, 1.49)**
Wild	Japan/ S Korea	**1.36[†] (0.12, 2.81)**	0.66	**1.46[§] (0.03, 3.82)**	0.41	0.33	0.40	0.28	-	0.60	0.40	0.43	0.39	0.68	**1.58[§] (0.21, 3.27)**
Wild	E China	**1.68[†] (0.31, 3.12)**	0.78	**3.05[§] 0.17, 5.76)**	**0.93[†] (0.14, 1.95)**	0.32	0.46	0.25	**1.16[‡] (0.17, 2.46)**	-	0.43	0.39	0.40	**0.80[†] (0.09, 1.61)**	**1.19[†] (0.17, 2.35)**
Wild	C China	0.42	0.52	0.46	0.47	0.37	0.24	0.35	0.52	0.79	-	0.36	0.35	0.53	0.77
Wild	SE Asia	0.31	0.29	0.36	0.37	0.38	0.35	0.45	0.32	0.32	0.30	-	0.43	0.29	0.39
Wild	Middle East	0.39	0.36	0.40	0.42	0.56	0.30	0.43	0.46	0.41	0.27	**0.67[§] (0.04, 1.62)**	-	0.42	0.56
Wild	Europe	0.17	0.18	0.22	0.20	0.15	**2.33[§] (0.53, 4.17)**	0.19	0.27	0.27	0.23	0.32	**0.61[§] (0.06, 1.26)**	-	0.35
Wild	North America	0.10	0.12	0.15	0.17	0.10	0.12	**0.41[§] (0.07, 0.86)**	0.12	0.28	0.21	0.10	0.09	**0.23[§] (0.01, 0.53)**	-

BSSV statistically supported migration rates with 95% Bayesian credible intervals (BCI), where the Bayes factor was >12. [†] 12 > BF ≥ 30; [‡] 30 > BF ≥ 100; [§] BF > 100
