# Supplementary figures and images for: Ecosystem Interactions Underlie the Spread of Avian Influenza A Viruses with Pandemic Potential
Source: PLoS Pathog. 2016 May 11;12(5):e1005620. doi: 10.1371/journal.ppat.1005620 (PMC4864295; doi:10.1371/journal.ppat.1005620)

Figure S1

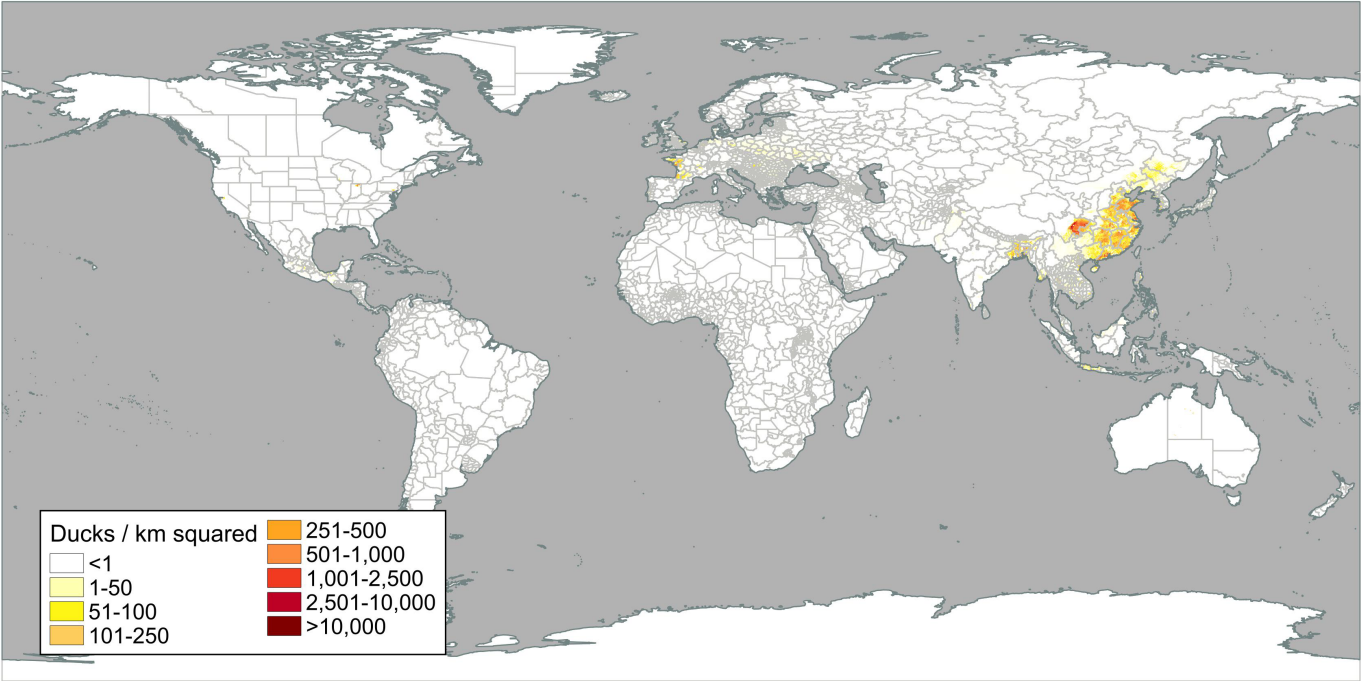

Supplement: S1 Fig — (PDF) [file ppat.1005620.s007.pdf]

Figure S2

A

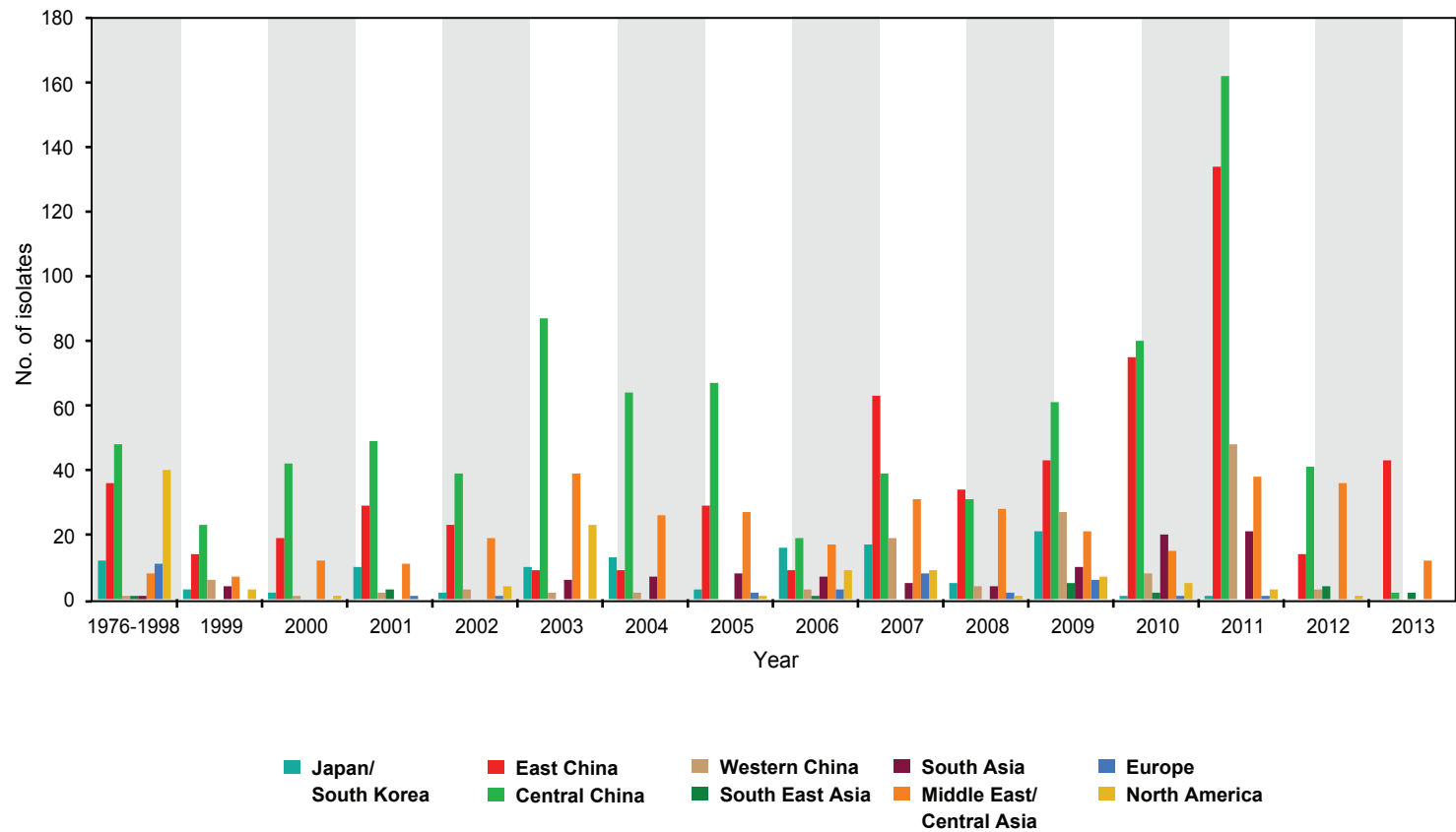

B

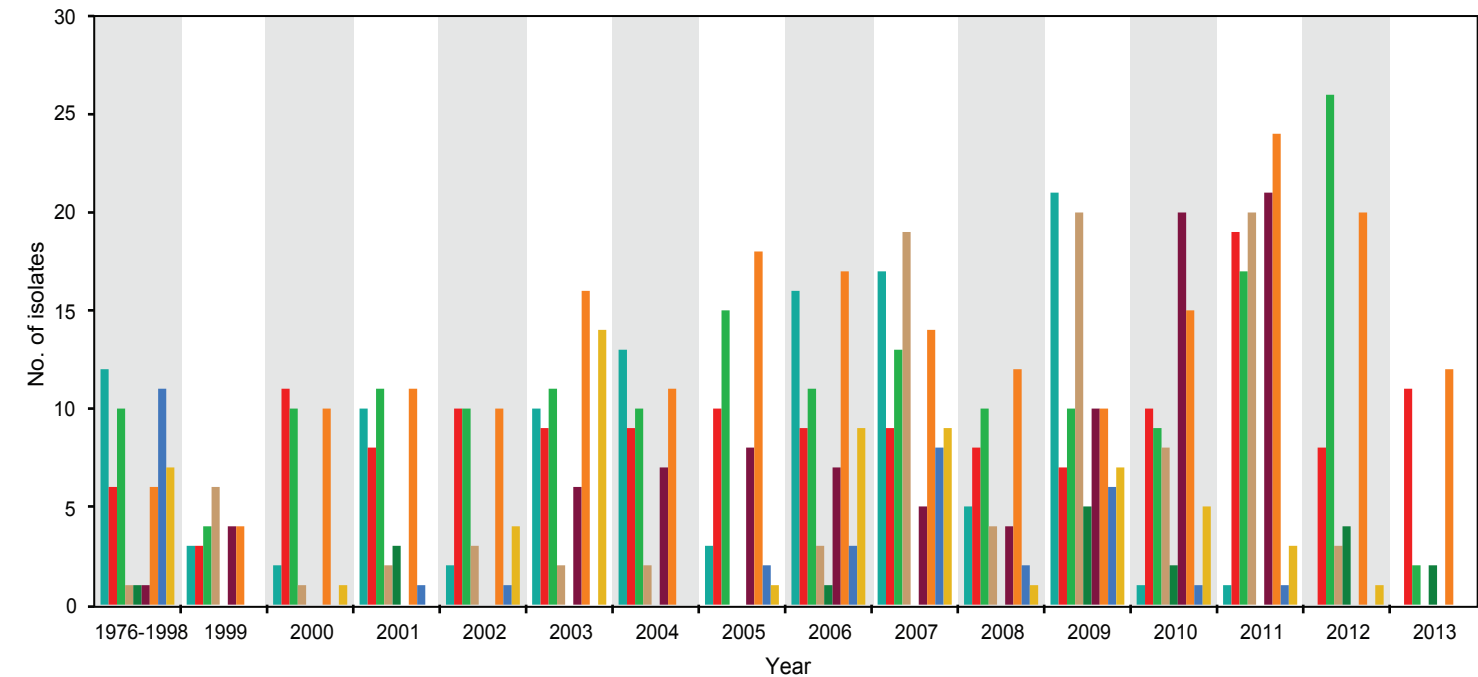

Supplement: S2 Fig — (A) Histogram of H9 avian influenza isolates included in the full dataset per year by regions. (B) Histogram of H9 avian influenza isolates following subsampling included in the final dataset per year by regions. (PDF) [file ppat.1005620.s008.pdf]

Figure S3

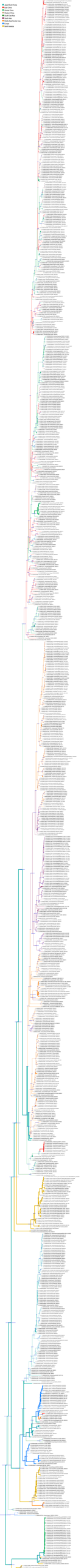

Supplement: S3 Fig — Purple bars on nodes indicate 95% Bayesian credibility intervals of divergence time estimates. (PDF) [file ppat.1005620.s009.pdf]

Figure S4

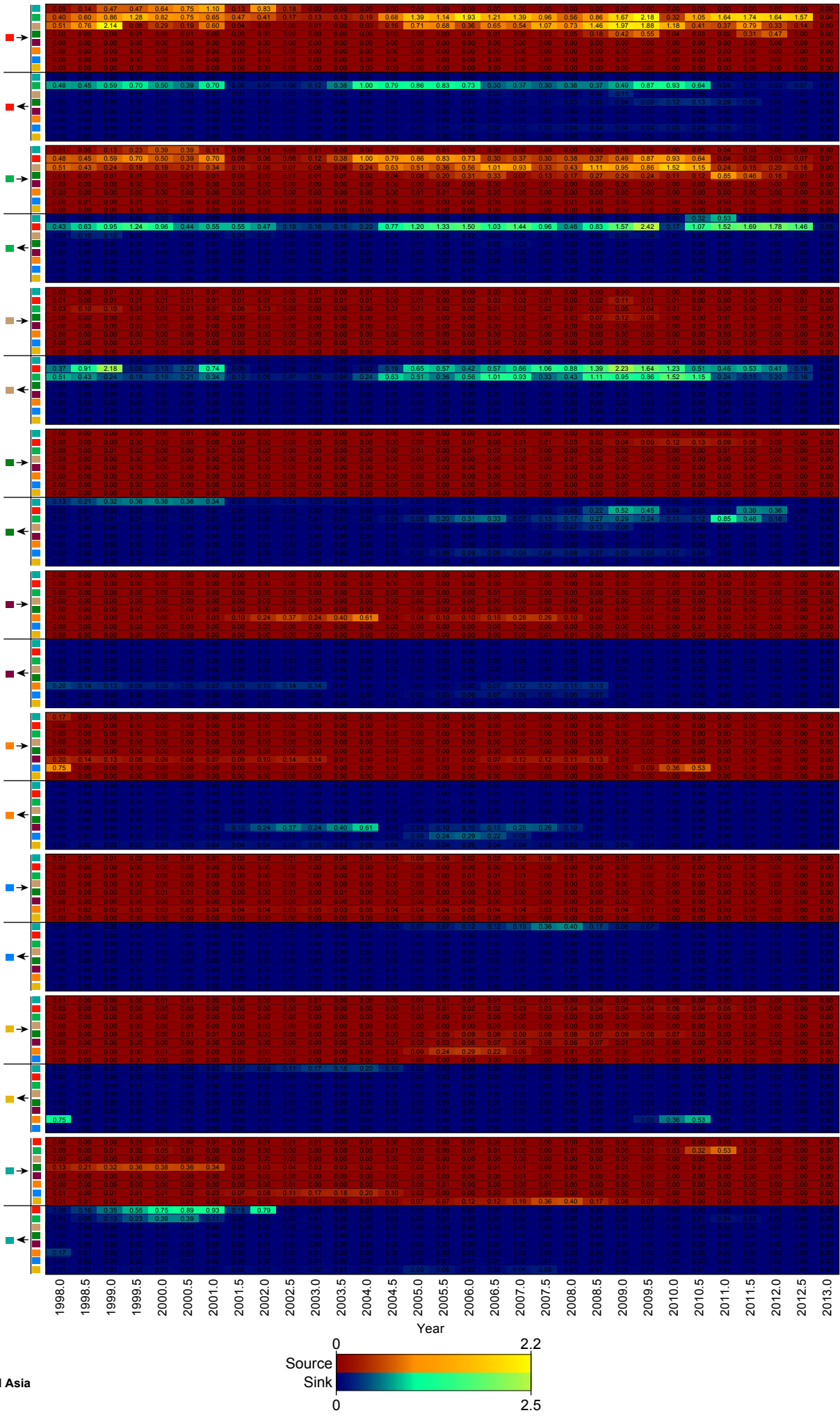

Supplement: S4 Fig — (PDF) [file ppat.1005620.s010.pdf]

Figure S5

A

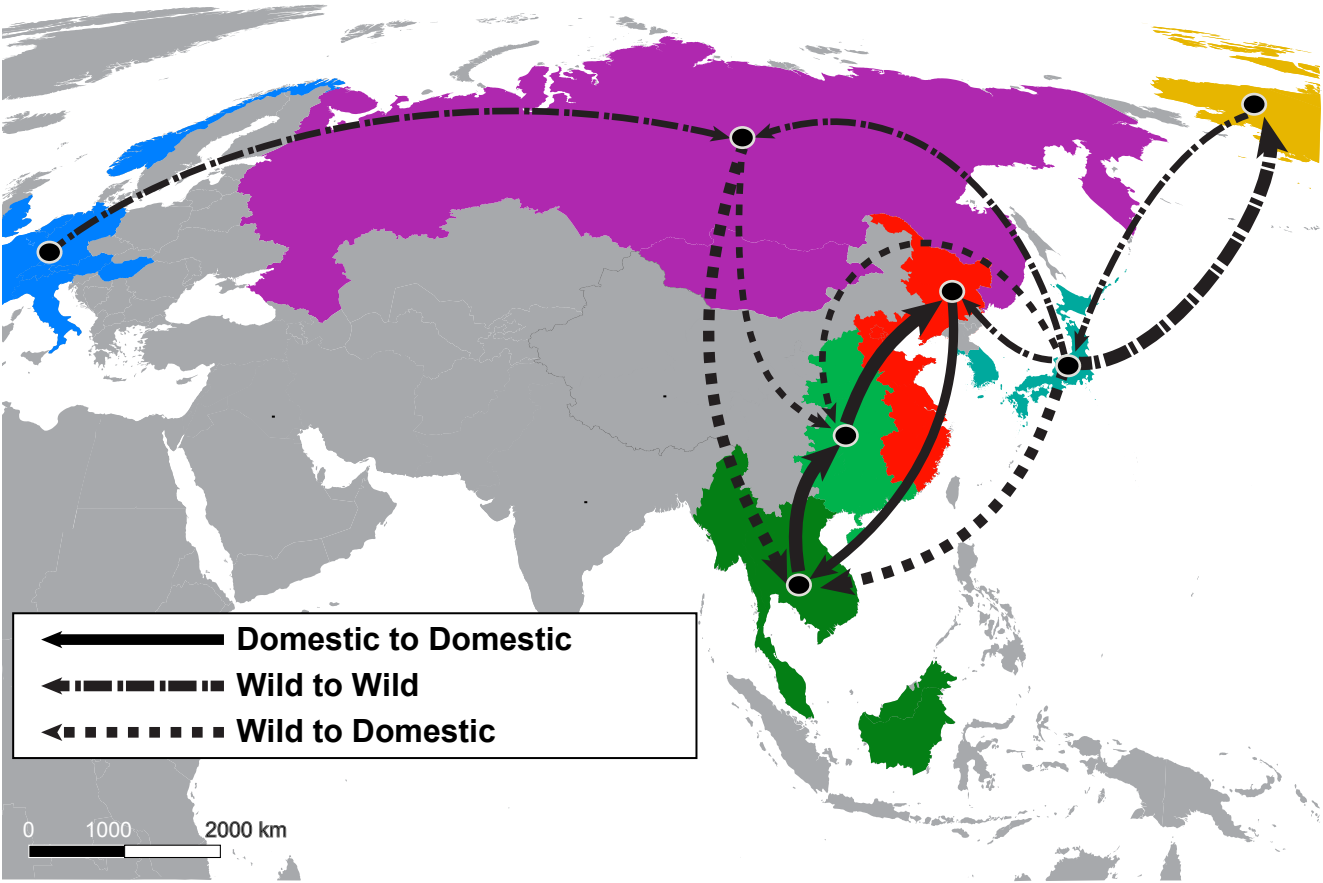

B

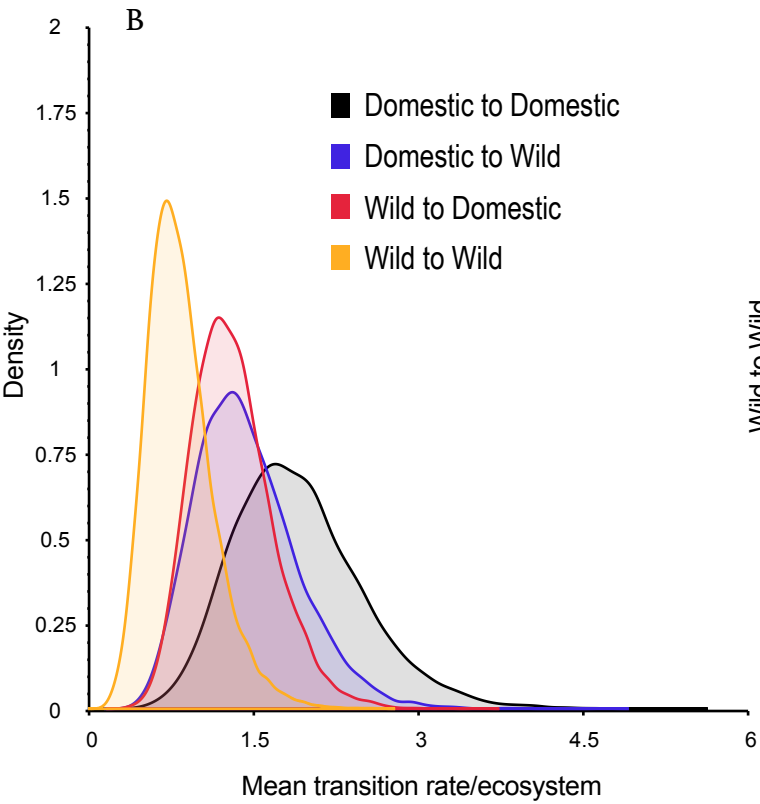

C

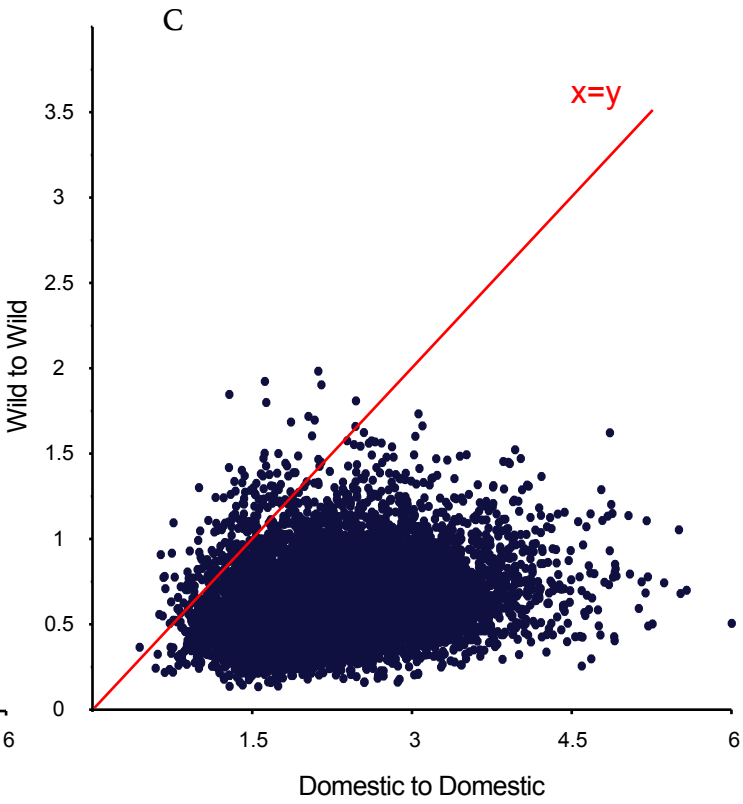

Supplement: S5 Fig — (A) Map showing statistically supported transitions between geographic regions by ecosystem. Line thickness corresponds to viral flow rates shown in S3 Table (thinnest <0.5; 0.5 to <1; 1 to <2; ≥2 thickest). (B) Density distribution of statistically supported mean transition rates between ecosystems. (C) Statistically supported mean migration rates per MCMC step of wild-to-wild avian transitions versus domestic-to-domestic avian transitions. (PDF) [file ppat.1005620.s011.pdf]

Figure S6

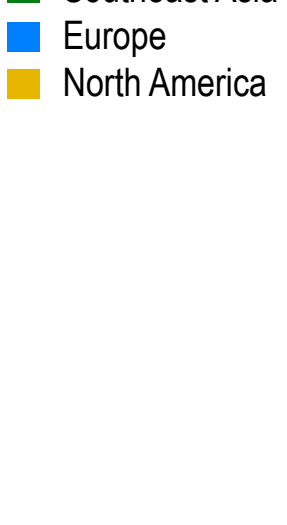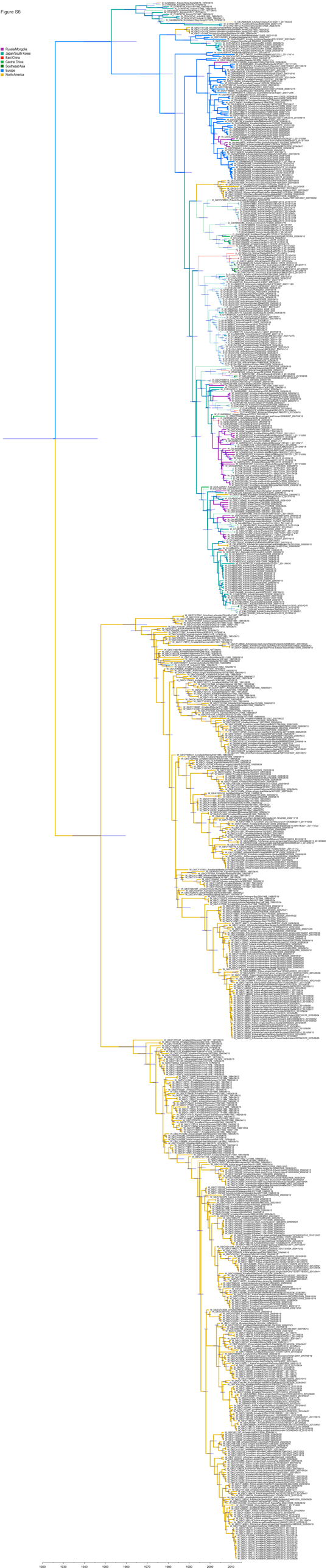

Supplement: S6 Fig — Purple bars on nodes indicate 95% Bayesian credibility intervals of divergence time estimates. (PDF) [file ppat.1005620.s012.pdf]

Figure S7

A

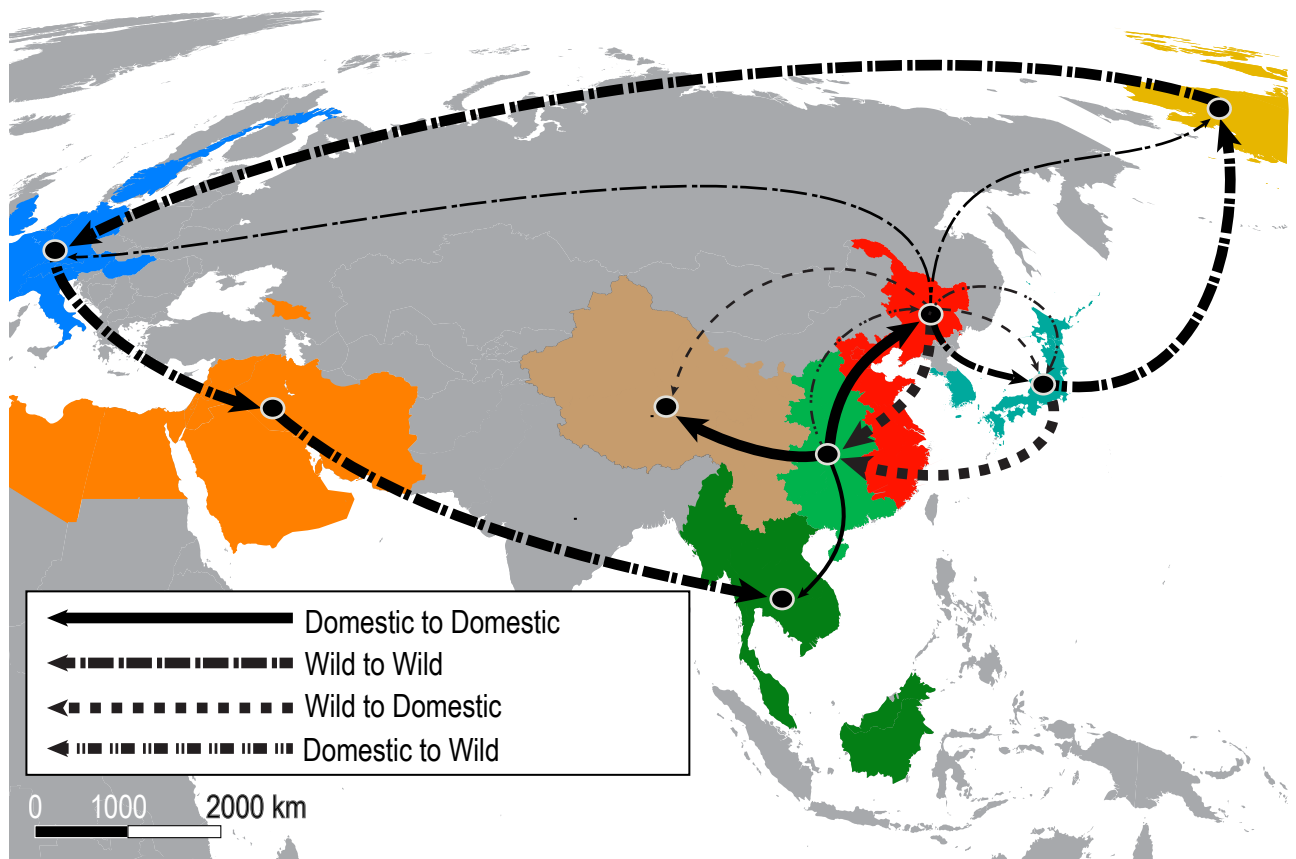

B

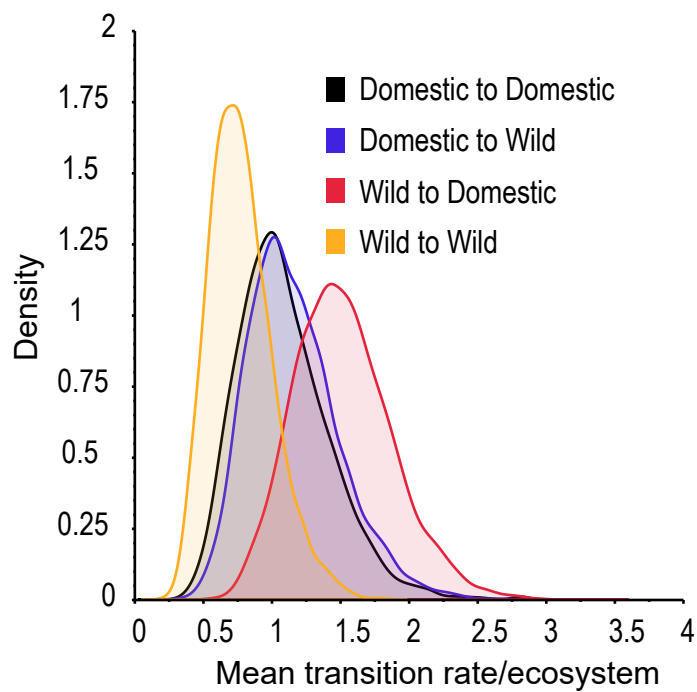

C

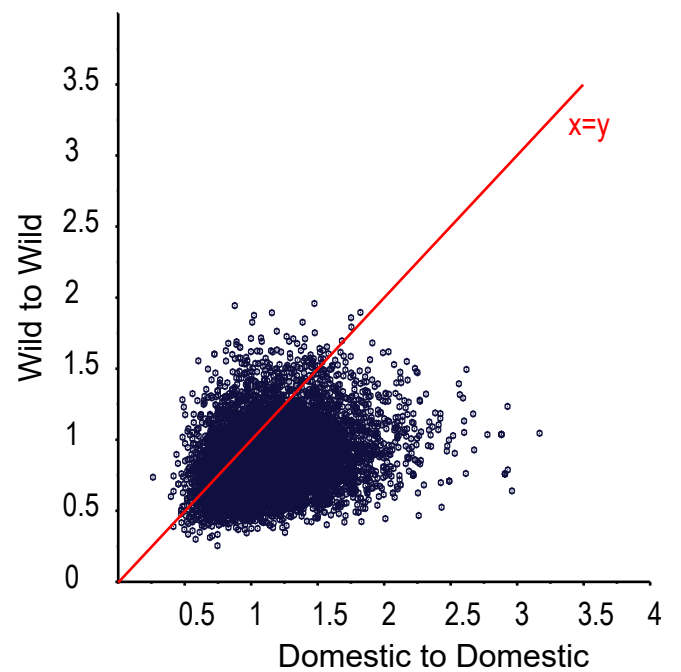

Supplement: S7 Fig — (A) Map showing statistically supported transitions between geographic regions by ecosystem. Line thickness corresponds to viral flow rates shown in S4 Table (thinnest <0.5; 0.5 to <1; 1 to <2; ≥2 thickest). (B) Density distribution of statistically supported mean transition rates between ecosystems. (C) Statistically supported mean migration rates per MCMC step of wild-to-wild avian transitions versus domestic-to-domestic avian transitions. (PDF) [file ppat.1005620.s013.pdf]

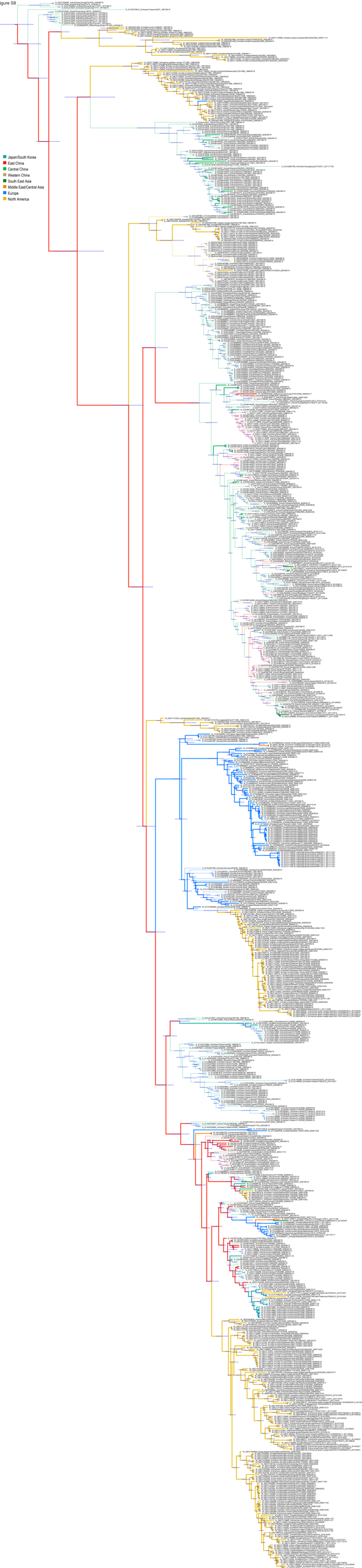

Supplement: S8 Fig — Purple bars on nodes indicate 95% Bayesian credibility intervals of divergence time estimates. (PDF) [file ppat.1005620.s014.pdf]

Figure S9

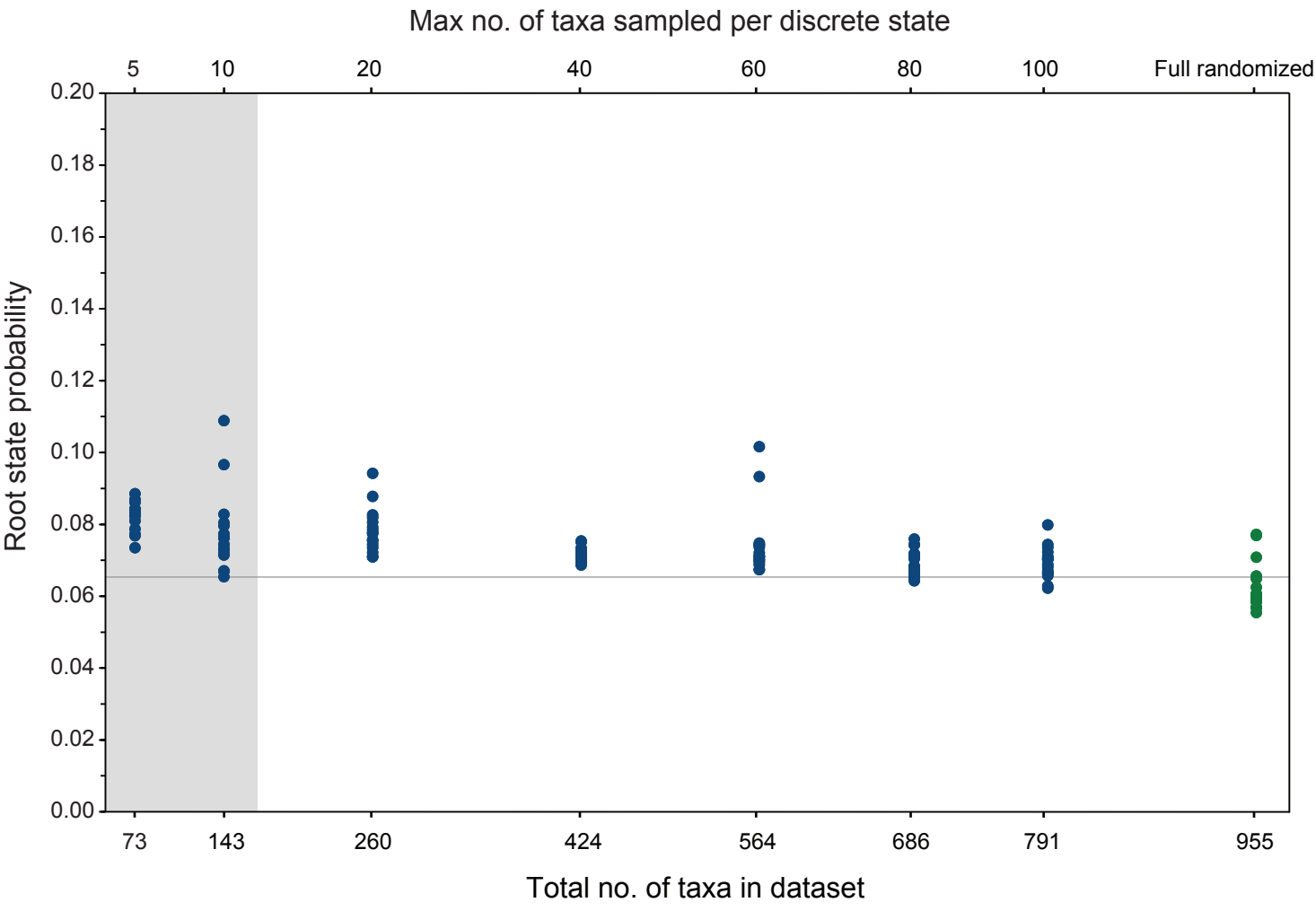

Supplement: S9 Fig — The final dataset is shown in green and alternative state sampling procedures indicated in blue. Grey line indicates the prior expectation for the root location probability. Shaded area indicates empirical posterior probability under conditions of over-parametization (i.e. more parameters estimated than data points observed). (PDF) [file ppat.1005620.s015.pdf]

Figure S10

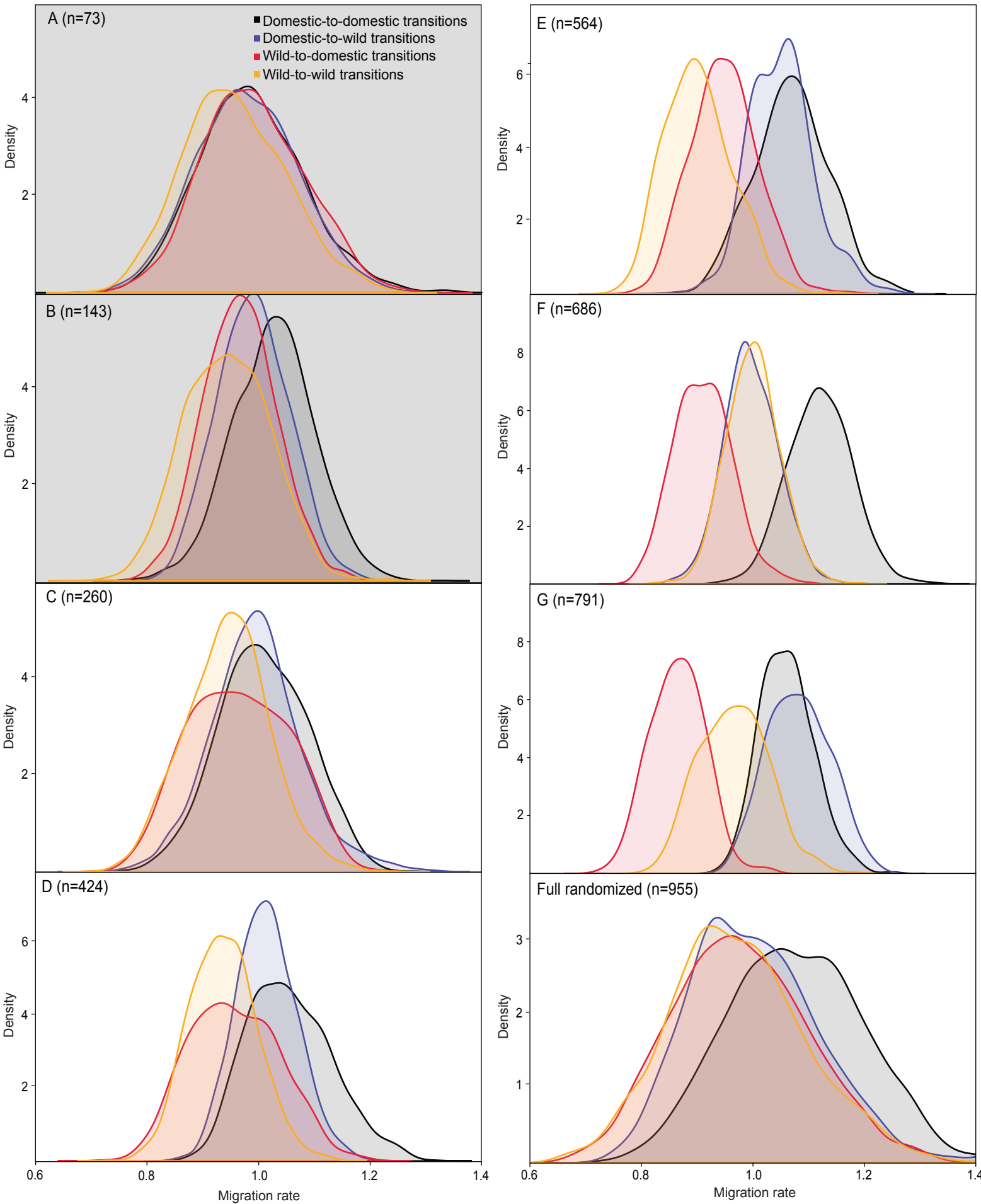

Supplement: S10 Fig — The number of sequenced used in each analysis were A) n = 73; B) n = 143; C) n = 260; D) n = 424; E) n = 564; F) n = 686; G) n = 791. (PDF) [file ppat.1005620.s016.pdf]

Figure S11

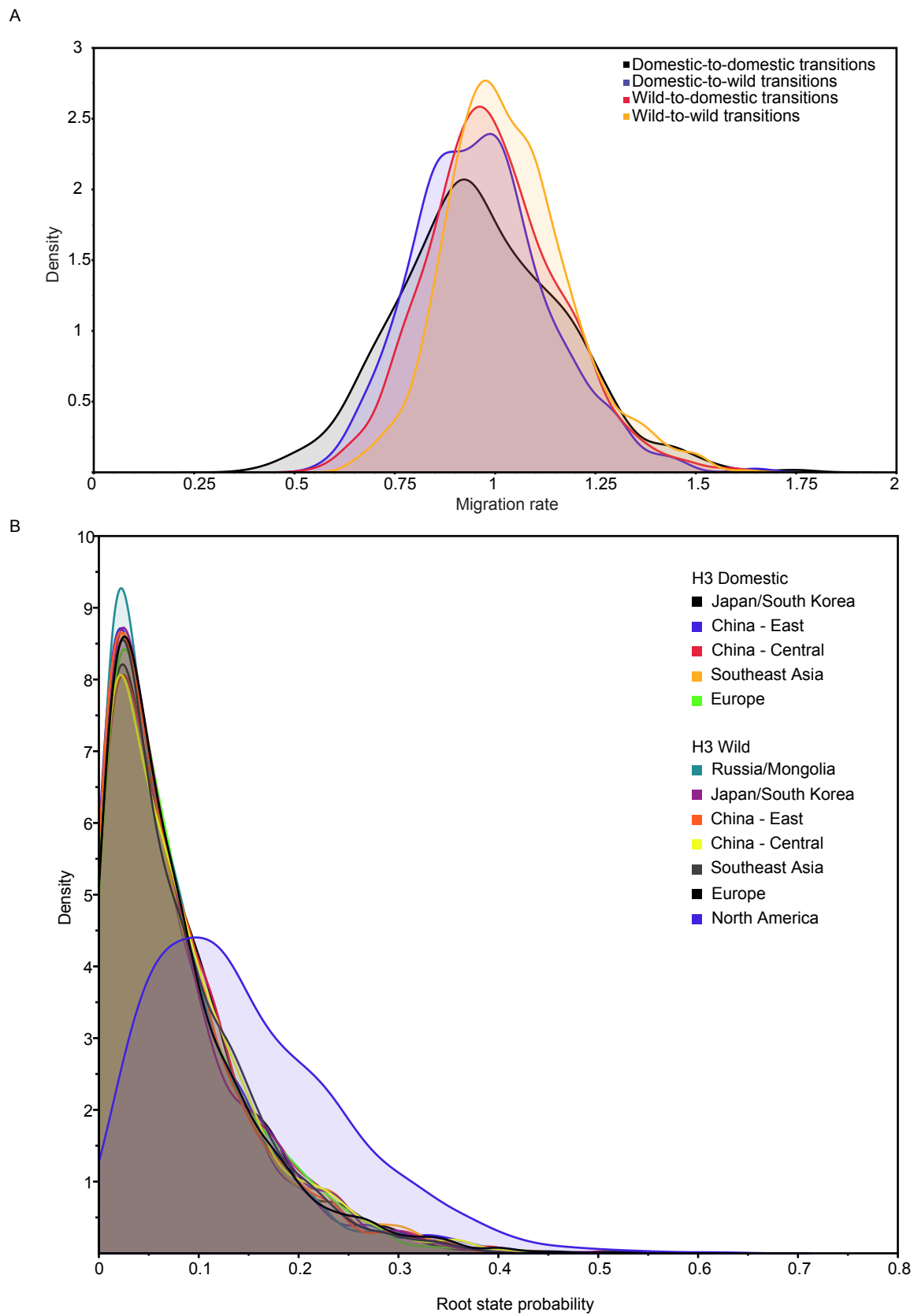

Supplement: S11 Fig — (PDF) [file ppat.1005620.s017.pdf]

Figure S12

A

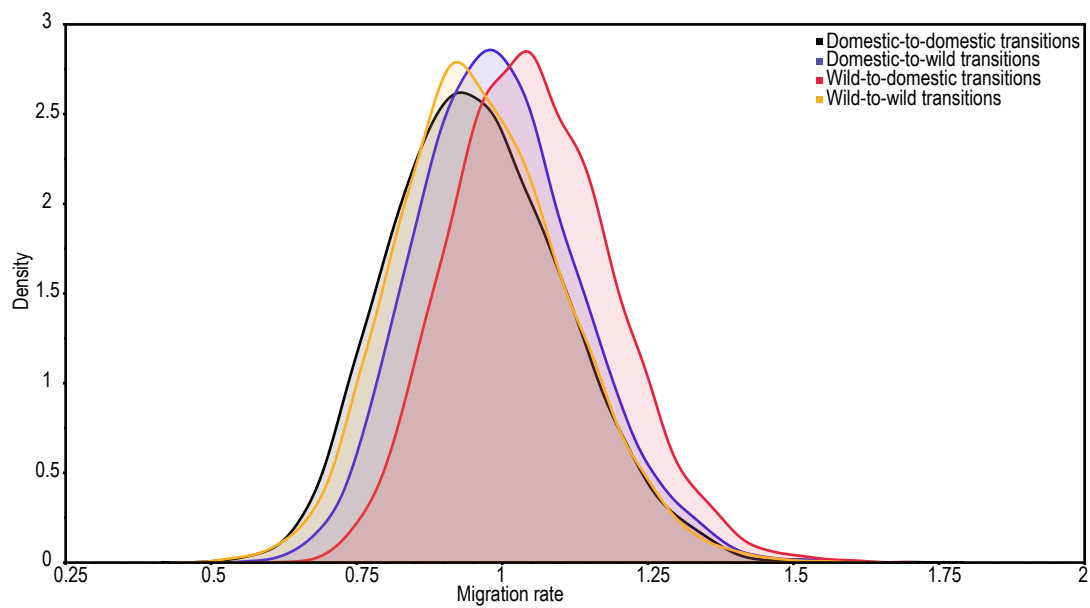

B

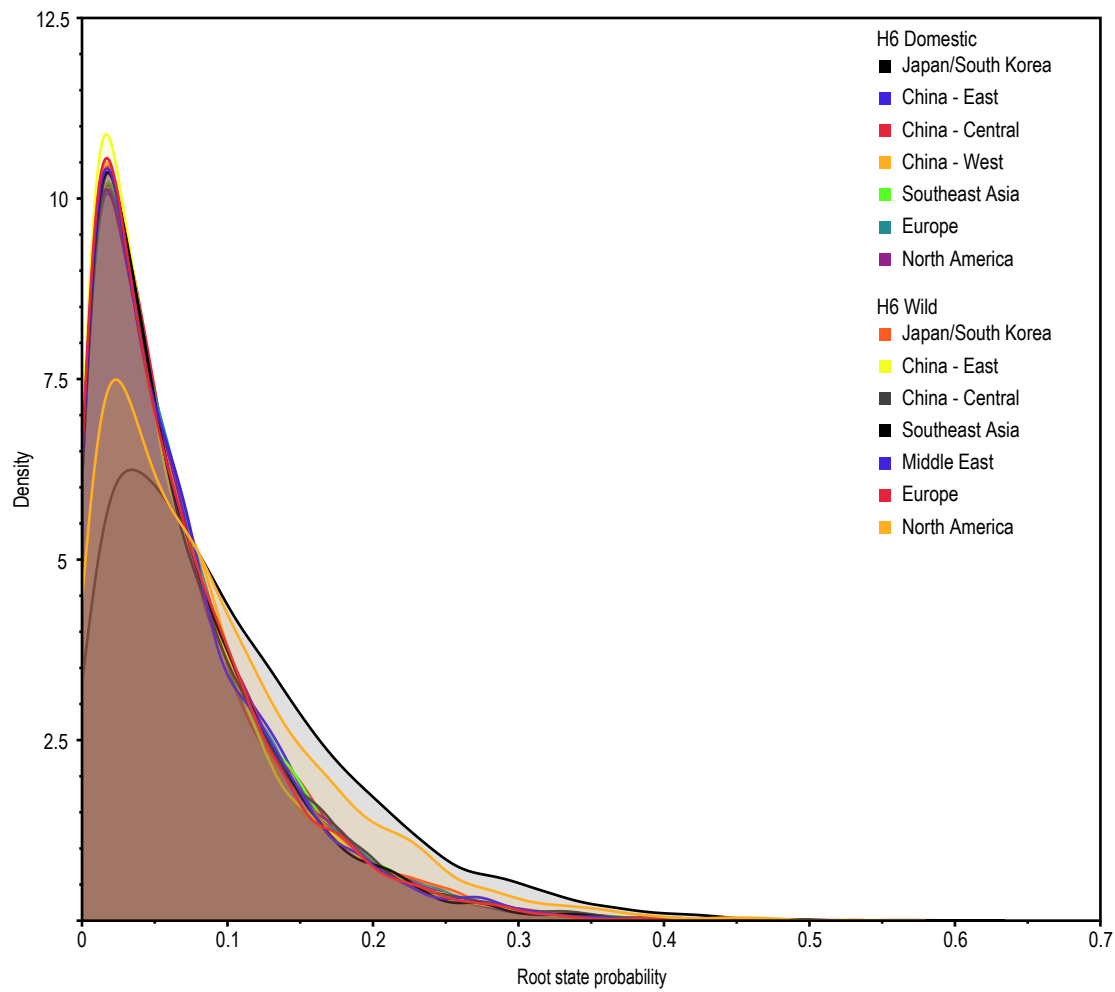

Supplement: S12 Fig — (PDF) [file ppat.1005620.s018.pdf]
